# The extracellular matrix proteoglycan lumican improves survival and counteracts cardiac dilatation and failure in mice subjected to pressure overload

**DOI:** 10.1038/s41598-019-45651-9

**Published:** 2019-06-24

**Authors:** Naiyereh Mohammadzadeh, Ida G. Lunde, Kine Andenæs, Mari E. Strand, Jan Magnus Aronsen, Biljana Skrbic, Henriette S. Marstein, Caroline Bandlien, Ståle Nygård, Joshua Gorham, Ivar Sjaastad, Shukti Chakravarti, Geir Christensen, Kristin V. T. Engebretsen, Theis Tønnessen

**Affiliations:** 1 Institute for Experimental Medical Research, Oslo University Hospital and University of Oslo, Oslo, Norway; 2 KG Jebsen Center for Cardiac Research, University of Oslo and Center for Heart Failure Research, Oslo University Hospital, Oslo, Norway; 3 Center for Molecular Medicine Norway, Oslo University Hospital and University of Oslo, Oslo, Norway; 4 Department of Genetics, Harvard Medical School, Boston, MA USA; 5 Bjørknes College, Oslo, Norway; 6 Department of Cardiothoracic Surgery, Oslo University Hospital, Oslo, Norway; 7 Department of Informatics, University of Oslo, Oslo, Norway; 8 Department of Medicine, Johns Hopkins University, Baltimore, PhD USA; 9 Present Address: Department of Ophthalmology and Pathology, NYU Langone Health, Alexandria Life Sciences Center, West Tower, New York, NY NY10011 USA; 10 Department of Surgery, Vestre Viken Hospital, Drammen, Norway

**Keywords:** Developmental biology, Disease model

## Abstract

Left ventricular (LV) dilatation is a key step in transition to heart failure (HF) in response to pressure overload. Cardiac extracellular matrix (ECM) contains fibrillar collagens and proteoglycans, important for maintaining tissue integrity. Alterations in collagen production and cross-linking are associated with cardiac LV dilatation and HF. Lumican (LUM) is a collagen binding proteoglycan with increased expression in hearts of patients and mice with HF, however, its role in cardiac function remains poorly understood. To examine the role of LUM in pressure overload induced cardiac remodeling, we subjected LUM knock-out (LUMKO) mice to aortic banding (AB) and treated cultured cardiac fibroblasts (CFB) with LUM. LUMKO mice exhibited increased mortality 1–14 days post-AB. Echocardiography revealed increased LV dilatation, altered hypertrophic remodeling and exacerbated contractile dysfunction in surviving LUMKO 1–10w post-AB. LUMKO hearts showed reduced collagen expression and cross-linking post-AB. Transcriptional profiling of LUMKO hearts by RNA sequencing revealed 714 differentially expressed transcripts, with enrichment of cardiotoxicity, ECM and inflammatory pathways. CFB treated with LUM showed increased mRNAs for markers of myofibroblast differentiation, proliferation and expression of ECM molecules important for fibrosis, including collagens and collagen cross-linking enzyme lysyl oxidase. In conclusion, we report the novel finding that lack of LUM attenuates collagen cross-linking in the pressure-overloaded heart, leading to increased mortality, dilatation and contractile dysfunction in mice.

## Introduction

Left ventricular (LV) pressure overload, as seen in patients with aortic stenosis and hypertension, leads to concentric LV remodeling. Cardiac remodeling encompasses functional and structural changes in cardiomyocytes, cardiac fibroblasts, and the extracellular matrix (ECM)^1^. Over time, and if not adequately treated, remodeling might progress into LV dilatation and failure^2^. The fundamental mechanisms underlying transition to dilatation and failure are not well known.

The cardiac interstitial ECM is composed of fibrillar collagens, elastic fibers and proteoglycans. The collagen network of the cardiac ECM provides the structural integrity of the heart, important for cardiac function and structure^3^. LV chamber stiffness and remodeling is influenced by cardiac collagen accumulation and cross-linking in experimental models of pressure overload^4,5^, as well as in patients with HF^5^. Upon pressure overload, cardiac fibrosis is a major operating pathophysiological mechanism affecting tissue architecture, electrical conduction, and diastolic and systolic properties of the myocardium^6^. A better understanding of the molecular players orchestrating the fibrotic pathways is essential to improve treatment options for patients.

Proteoglycans are macromolecules composed of a core protein substituted with covalently linked glycosaminoglycan (GAG) chains, and are major constituents of the ECM^7^. Cell surface proteoglycans, i.e. syndecans and glypicans, are important to pressure overload driven cardiac remodeling processes, including hypertrophic growth, fibrosis and inflammation^8–11^. Lumican (LUM) is a keratan sulfate small leucine-rich proteoglycan (SLRP) localized to the ECM, and known to regulate collagen fibrillogenesis in connective tissues, e.g. cornea, tendon and skin^12,13^. LUM binds fibrillar collagens^14^, and regulates collagen fibril thickness and interfibrillar spacing, important for tissue integrity and corneal transparency^12,15^. LUM is central to development of pulmonary and hepatic fibrosis^16,17^, is abundant in fibrotic tissues including the thickened intima of human atherosclerotic coronary arteries^18^, and is present in the developing myocardium^19,20^. Transforming growth factor (TGF)β, a major pro-fibrotic growth factor, is believed to interact with LUM^21,22^. Although a profibrotic effect of LUM in other organs has been reported, the role of LUM in cardiac fibrosis remains largely unknown. LUM is also known to be involved in inflammation^23^. It regulates immune cell recruitment after corneal injury^24,25^, and promotes toll-like receptor 4 (TLR4)-mediated innate immune responses^26^. Its role in the cardiac immune response during pressure overload-induced remodeling is not well known.

Our group has previously shown that LUM levels are increased in hearts of mice and patients with heart failure^27^. Here, we investigated the hypothesis that LUM is important for cardiac remodeling, fibrosis and inflammation following experimental pressure overload of the heart. LUM knock-out (LUMKO) mice were subjected to aortic banding (AB) *in vivo*, and cultured cardiac fibroblasts were treated with LUM *in vitro*.

## Results

### Reduced survival of LUMKO mice

Genotyping (Fig. S1A), mRNA and protein analyses of hearts of mice (Fig. S1B,C, respectively), confirmed the LUMKO genotype. We found no compensatory changes in cardiac FMOD expression (Fig. S1D), a keratan sulfate SLRP binding to the same region of collagen as LUM^28^ and with redundant roles in fibrillogenesis^29^. Intercrosses of heterozygous LUMKO were set up to generate LUMKO and WT littermates; deviations from the expected Mendelian distribution indicated reduced perinatal survival (i.e. 8 out of 236 pups were LUMKO (3%)) (Table S1).

### LUMKO mice show no overt cardiac phenotype without stress

To investigate whether lack of LUM affected cardiac morphology and function, baseline echocardiography was performed in adult LUMKO mice and WT littermate controls. We observed no differences in cardiac dimensions and function (Table S2). In line with this, we found no differences in heart or lung weight or the expression of cardiac signature molecules of heart failure, ANP and BNP (encoding atrial and brain natriuretic peptides, respectively), pathological remodeling (increased myosin heavy chain β/α ratio, encoded by MYH7 and MYH6, respectively) and hypertrophy (ACTA1 encoding α-skeletal actin) (Table S2). Thus, LUMKO mice showed no overt cardiac phenotype without stress.

### Increased mortality in LUMKO mice following pressure overload

**Figure 1:**
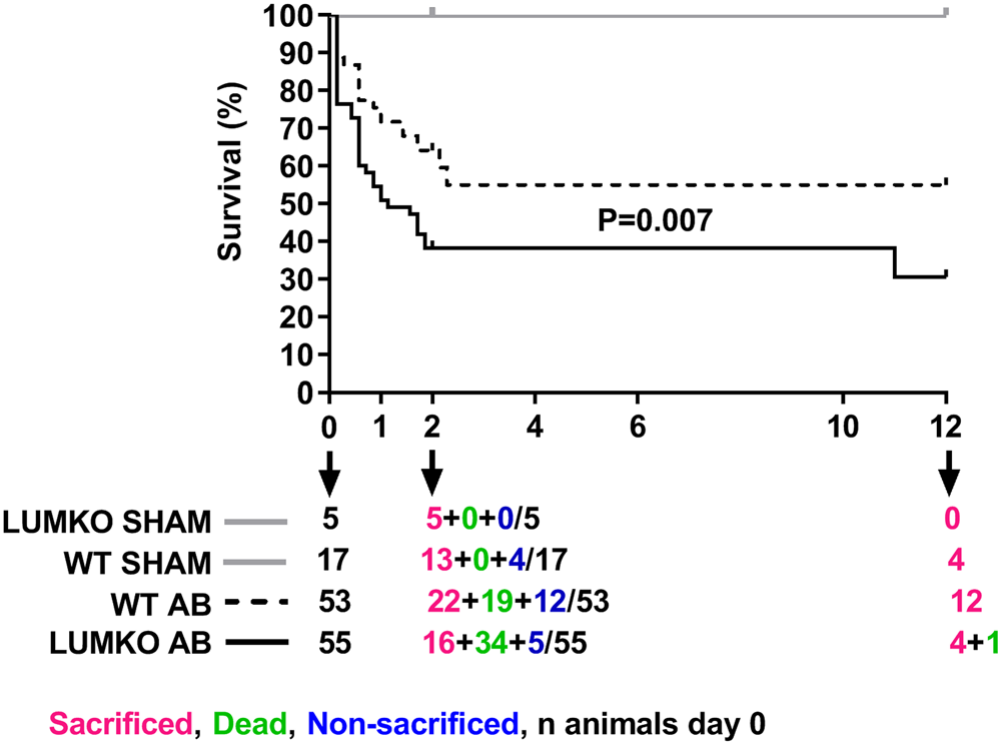
Increased mortality of LUMKO mice post-AB. Kaplan-Meier survival curves for lumican knock-out (LUMKO, n = 55) and wild-type (WT, n = 53) mice 12 weeks post-aortic banding (AB). We had no mortality in SHAM groups. At 2w, we sacrificed 5 LUMKO SHAM and 13 WT SHAM. The remainder 4 WT SHAM lived until 12w. However, we had significant mortality in AB groups. At 2w, in WT AB group (n = 53), we sacrificed 22 mice, 19 mice died up until 2w and 12 mice lived until 12w (22 + 19 + 12 = 53). At 2w, in LUMKO AB group, we sacrificed 16 mice, 34 mice died up until 2w and 5 mice lived until 12w (one died after 10w) (16 + 34 + 5 = 55). Differences were tested using Log-rank (Mantel–Cox) test, p = 0.007.

LUM mRNA was increased in hearts of WT mice post-AB (Fig. S1E), confirming our previous results^27^. To investigate the role of LUM in cardiac remodeling following pressure overload, LUMKO mice were subjected to pressure overload by AB. Importantly, the Kaplan-Meier survival curve revealed an increased mortality in LUMKO mice compared to WT controls post-AB (Fig. 1). At 24 h post-AB, 24% of LUMKO mice had died, compared to 12% of the WTs, and at 2 weeks post-AB, 62% of LUMKO mice were dead, compared to 36% of controls. The mortality rate was similar in both genotypes from 3 to 12 weeks post-AB. We observed no mortality in SHAM-operated mice.

### Increased dilatation, altered hypertrophic remodeling and exacerbated contractile dysfunction in LUMKO mice following pressure overload

**Figure 2:**
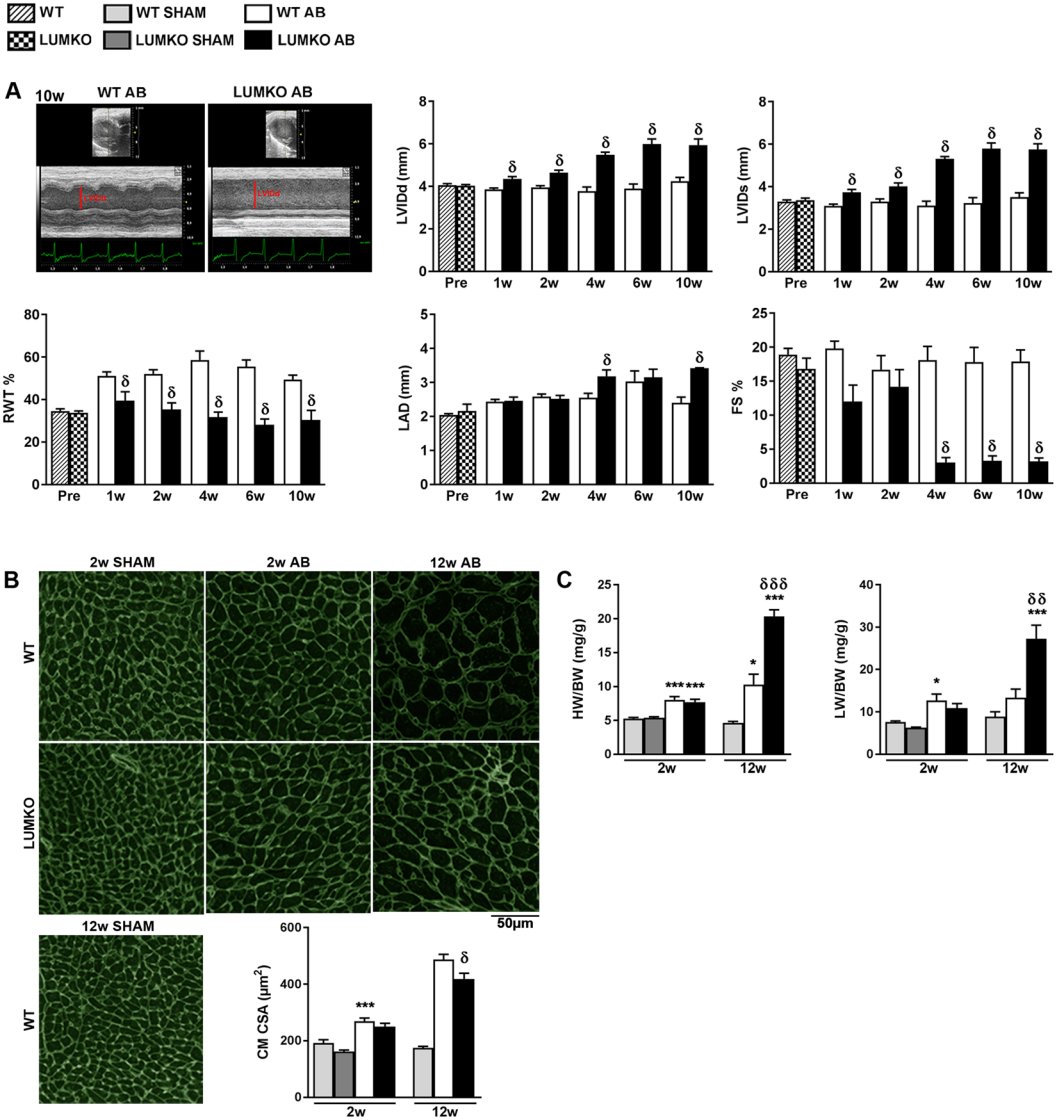
Increased left ventricular dilatation and exacerbated contractile dysfunction in LUMKO mice post-AB. (**A**) Serial echocardiography of lumican knock-out (LUMKO, n = 3–17) and wild-type (WT, n = 16–35) pre- and 1–10 weeks (w) post-aortic banding (AB), showing left ventricular (LV) internal diameter in diastole (LVIDd), and systole (LVIDs), and relative wall thickness (RWT %), and left atrial diameter (LAD) and fractional shortening (FS %). Representative 2D and M-mode echocardiograms are shown in A. (**B**) Representative Wheat Germ Agglutinate (WGA)-stained mid-ventricular sections and quantitative measurement of cardiomyocyte cross-sectional areas (CSA) in LUMKO and WT 2w and 12w post-SHAM and -AB (n SHAM 3–5, n AB 4–10, n = 2000–15000 cardiomyocytes). Scale bars 50 µm. (**C**) Heart and lung weights normalized to body weight (HW/BW and LW/BW, respectively) in LUMKO and WT 2w and 12w post-SHAM and -AB (n SHAM 3–12, n AB 11–20). The data are presented as mean ± SEM. Differences were tested using one-way ANOVA with Dunn’s post-hoc test vs. WT SHAM, ***p < 0.005; **p < 0.01; *p < 0.05, or one-way ANOVA with Tukey’s multiple comparisons test vs. WT AB, δp < 0.05; δδp < 0.01; δδδp < 0.005 (**B**,**C** 2w and 12w), or two-way ANOVA, δp < 0.05 (**A**).

Serial echocardiography was performed in LUMKO mice at 1, 2, 4, 6 and 10 weeks post-AB. Hearts were harvested at 2 and 12 weeks post-AB for molecular analyses. Interestingly, increased LV dilatation was evident in LUMKO compared to WT mice from 1 to 10 weeks post-AB, assessed as the LV internal diameter in diastole (LVIDd) and systole (LVIDs) (Fig. 2A). Thickness of the interventricular septum and LV posterior wall in diastole (IVSd and LVPWd, respectively) were reduced in LUMKO vs. WT mice post-AB (Table S2), and thus, the relative wall thickness (RWT %, calculated from LVPWd + IVSd/LVIDd) was reduced in LUMKO from 1 to 10 weeks post-AB (Fig. 2A). Histology of mid-ventricular sections showed that the cross-sectional area (CSA) of LUMKO cardiomyocytes was smaller than that of WT 12w post-AB (Fig. 2B), consistent with the reduced wall thickness observed on echocardiograms. There was no difference in cardiomyocyte cross sectional area (CSA) between LUMKO and WT 2w post-AB (Fig. 2B). The exacerbated cardiac remodeling of LUMKO vs. WT mice was evident also from the increased heart weight at 12 weeks post-AB (Fig. 2C) and LAD at 4 and 10 weeks post-AB (Fig. 2A). Consistent with exacerbated heart failure in LUMKO mice post-AB, lung weight was increased in LUMKO compared to WT mice at 12 weeks post-AB (Fig. 2C), and reduced cardiac contractility was evident by reduced LV fractional shortening in LUMKO mice vs. WT 4 to 10 weeks post-AB (Fig. 2A). Despite the differences in phenotype observed on echocardiography and heart and lung weights, the expression of signature heart failure markers ANP, BNP, growth differentiation factor GDF15 and ratio of hypertrophic molecules MYH7/MYH6 were not different in LUMKO and WT hearts 2 and 12 weeks post-AB (Fig. S2A–D, respectively). Moreover, there were no major differences in the hemodynamic parameters between the two genotypes at any time-points post-AB (Table S3).

RNA sequencing was performed on LUMKO and WT LVs 2 weeks post-AB. 714 differentially expressed (DE) transcripts were identified, i.e. 526 > 1.33-fold up- and 188 < 0.75-fold down-regulated, (p < 0.001). Consistent with a role for LUM in cardiac remodeling and failure, Ingenuity pathway analysis (IPA) of DE transcripts identified 11 cardiotoxicity pathways including “cardiac infarction”, “congenital heart anomaly”, “cardiac hypertrophy”, “cardiac dysfunction”, “cardiac necrosis/cell death”,”cardiac fibrosis”, “cardiac congestive/cardiac failure/heart failure”, “cardiac arrhythmia”, “cardiac output”, “cardiac enlargement” and “cardiac inflammation” (Table S4), consistent with our *in vivo* cardiac phenotype.

### Reduced collagen cross-linking and expression of markers of myofibroblast transdifferentiation in LUMKO hearts following pressure overload

**Figure 3:**
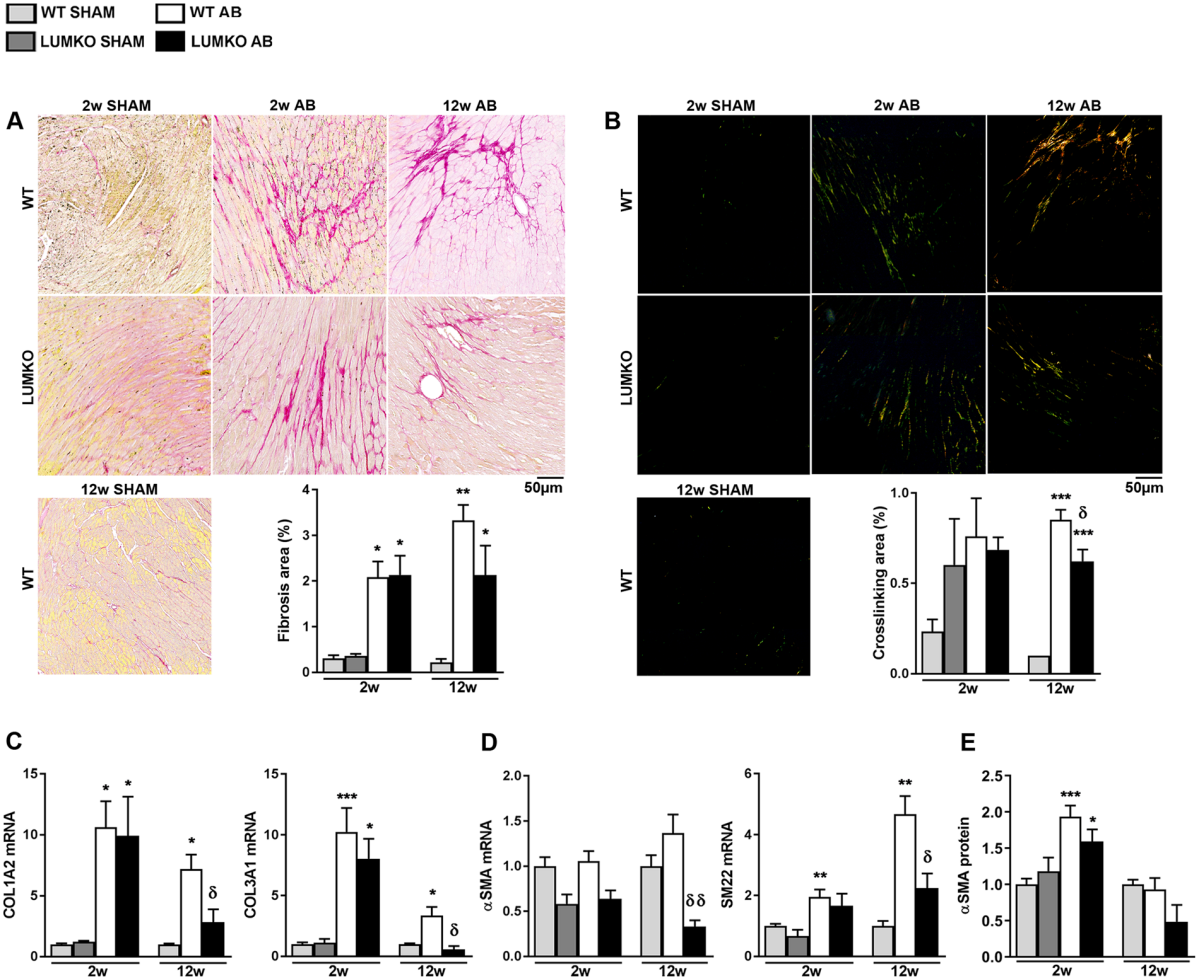
Reduced collagen cross-linking and collagen expression in hearts of LUMKO mice post-AB. Lumican knock-out (LUMKO) and wild-type (WT) mice were subjected to aortic banding (AB) for 12 weeks (w). (**A**,**B**) Representative images of mid-ventricular histology sections 2w and 12w post-SHAM and-AB, stained with Picrosirious Red visualizing fibrillar collagens (**A**, non-polarized light, bright field) and collagen cross-linking (**B**, polarized light). Fibrotic area = area of red staining/total area in %. Collagen crosslinking area = area of orange/green (cross-linked collagens)/total area in % (n SHAM 3–4, n AB 4–10). Scale bars 50 µm. (**C**,**D**) Relative left ventricular (LV) mRNA levels of fibrillar collagens I and III (COL1A2 and COL3A1), and myofibroblast differentiation marker αSMA and SM22 (n SHAM 4–14, n AB 3–22). mRNA expression was normalized to expression of ribosomal protein L32 (RPL32). (**E**) Quantitative protein levels of the myofibroblast differentiation marker alpha-smooth muscle actin (αSMA) in LVs of LUMKO and WT mice 2w and 12w post-SHAM and-AB (2w: n WT SHAM = 9, n WT AB = 13, n LUMKO SHAM = 9, n LUMKO AB = 14 and 12w: n WT SHAM = 4, n WT AB = 12, n LUMKO AB = 3). Cultured cardiac fibroblasts (CFB) were used as positive control. Coomassie staining was used as loading control. Immunoblots are presented in Fig. S5 (2w) and Fig. S6 (12w). The data are presented as mean ± SEM. Differences were tested using one-way ANOVA with Dunn’s post-hoc test vs. WT SHAM, *p < 0.05; **p < 0.01; ***p < 0.005; or an unpaired t-test vs. WT AB, δp < 0.01.

To examine whether the increased LV dilatation in LUMKO mice post-AB was associated with alterations in collagen deposition and cross-linking, Picrosirious red-stained mid-ventricular sections were examined under non-polarized and polarized light, respectively. Histology showed that there was no difference in total collagen deposition and collagen cross-linking between LUMKO and WT mice 2w post-AB (Fig. 3A,B). However, by 12w post-AB, total collagen deposition tended to be reduced in LUMKO vs. WT although not being statistically significant (p = 0.15, Fig. 3A). Importantly, collagen cross-linking was reduced in LUMKO vs. WT 12w post-AB (Fig. 3B) indicating loss of the structural integrity of the collagen matrix when LUM is lacking. We found no alteration in the mRNA levels of the collagen cross-linking enzyme LOX (Fig. S2E). We did, however, find reduced mRNA expression of COL1A2 and COL3A1 in LUMKO hearts vs. WT 12w post-AB (Fig. 3C). Moreover, mRNA levels of the myofibroblast differentiation markers α-smooth muscle actin (αSMA) and SM22 were reduced in LUMKO hearts vs. WT 12w post-AB (Fig. 3D). On the protein level there was a tendency to reduced levels of αSMA at 2 and 12 weeks (p = 0.14 and 0.23, respectively) (Figs 3E and S5, S6). Taken together, collagen cross-linking and mRNA levels of myofibroblast transdifferentiation markers were reduced in LUMKO hearts accompanied by a reduction in collagen expression in response to pressure overload *in vivo*.

Transcriptional profiling of the 714 DE transcripts from RNA-sequencing of LUMKO and WT hearts 2 weeks post-AB was performed by gene ontology (GO) and KEGG pathway enrichment (Tables S5, S6 respectively). We identified 69 enriched gene ontology (GO) categories (Table S5). Interestingly, and consistent with a role for LUM in regulation of the cardiac ECM and inflammation, “extracellular space”, “inflammatory response” “defense response” were the top three enriched GO categories. Supporting a role for LUM in cardiac ECM regulation, 54 DE transcripts were in the “extracellular space”, 20 in the “extracellular matrix”, 89 in “extracellular region”, 15 in “proteinaceous extracellular matrix” and 83 in the “extracellular region part” categories. Many GO categories associated with inflammation and infection were enriched, as well as categories associated with responses to stress or stimulus, and cell signaling. We also identified four enriched KEGG pathways (Table S6); “Staphylococcus aureus infection”, “Complement and coagulation cascades”, “circadian rhythm – mammal” and “systemic lupus erythematosus”, i.e. inflammation-related. Thus, our transcriptomic profiling of LUMKO hearts post-AB was consistent with a role for LUM in cardiac ECM remodeling and inflammation *in vivo*.

### Lumican regulates mRNA levels of myofibroblast transdifferentiation markers, proliferation markers, and production of ECM molecules important for fibrosis in cardiac fibroblasts *in vitro*

**Figure 4:**
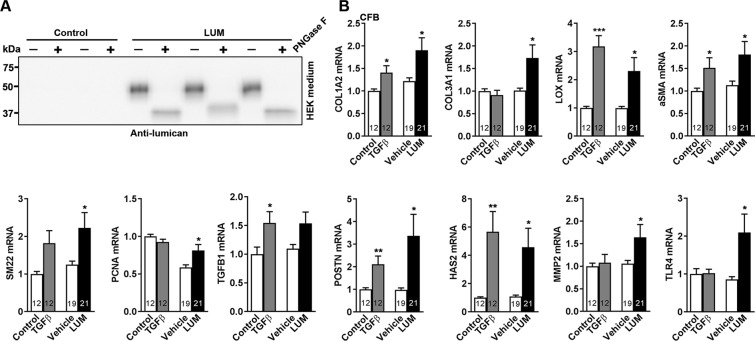
Increased mRNA expression of myofibroblast markers and ECM components including collagens in cardiac fibroblasts treated with LUM. Cultured cardiac fibroblasts from neonatal rats (n = 3 cell isolations) were treated with LUM or vehicle conditioned medium for 24 h. Non-treated cells (control) and cells treated with the pro-fibrotic growth factor transforming growth factor (TGF)β1 served as controls. (**A**) LUM was produced by transfection of human endothelial kidney (HEK)293 cells with human LUM. LUM was secreted into the cell medium as 50 kDa glycosylated proteoglycan (Fig. S8). PNGaseF treatment results in a 38 kDa deglycosylated core protein (Fig. S8). (**B**) mRNA expression of fibrillar collagens I and III (COL1A2 and COL3A1), the collagen cross-linking enzyme lysyl oxidase (LOX), the myofibroblast signature genes αSMA and SM22, the proliferating cell nuclear antigen (PCNA), TGFβ1 and the extracellular matrix components periostin (POSTN), hyaluronan synthase 2 (HAS2), matrix metalloproteinase 2 (MMP-2) and toll-like receptor-4 (TLR-4). mRNA expression was normalized to expression of ribosomal protein L32 (RPL32). Data are presented as mean ± SEM. Differences were tested using an unpaired t-test vs. Vehicle, *p < 0.05, or vs. Control, ***p ≤ 0.005; **p ≤ 0.01.

To investigate whether LUM had direct effects on cardiac fibroblast function, cultured cardiac fibroblasts from neonatal rats were treated with LUM *in vitro*. Human recombinant LUM was overexpressed in human endothelial kidney (HEK)293 cells, and LUM was secreted to the cell medium as 50 kDa glycosylated proteoglycan (Figs 4A and S3A,B). The conditioned medium was used to stimulate neonatal rat cardiac fibroblasts. The non-glycosylated LUM was not secreted into the medium, but found in the cell lysate at the predicted size (38 kDa) of the LUM core protein (Fig. S3A). The LUM treatment of cardiac fibroblasts did not lead to compensatory alterations in FMOD expression (Fig. S3C). Treatment of cells with conditioned medium from cells elicits an inflammatory response compared to non-treated fibroblasts (Fig. S3D). Thus, vehicle-treated cardiac fibroblasts were used as control for LUM treatment.

Importantly, treatment of neonatal rat cardiac fibroblasts with LUM revealed that expression of collagens I and III, i.e. COL1A2 and COL3A1 (Fig. 4B), and the collagen cross-linking enzyme LOX (Fig. 4B) was increased compared to vehicle control. Furthermore, expression of the myofibroblast signature molecules αSMA and SM22 (Fig. 4B) and the proliferation marker PCNA (encoding proliferating cell nuclear antigen) (Fig. 4B) on the mRNA level were increased by LUM treatment. Using a proliferation assay showed a tendency to an increase in cells after stimulation with LUM compared to vehicle (p = 0.13) (Fig. S7). mRNA levels of periostin (encoded by POSTN), a central ECM molecule and marker of ECM remodeling, was increased by LUM treatment (Fig. 4B). LUM also increased the mRNA levels of hyaluronan synthase 2 (HAS2) which facilitates the synthesis of hyaluronan, a component of the ECM important for remodeling and wound healing (Fig. 4B). Moreover, LUM up-regulated the expression of matrix metalloproteinase 2 (MMP-2), consistent with previous results (Fig. 4B)^27^. Finally, LUM increased the expression of toll-like receptor 4 (TLR-4), a transmembrane receptor known to be regulated by LUM (Fig. 4B). Thus, our findings collectively suggested that LUM regulates mRNA levels of molecules involved in structural integrity and composition of the cardiac ECM through a direct effect on cardiac fibroblasts.

### No alteration of immune cell infiltration in LUMKO mice following pressure overload

**Figure 5:**
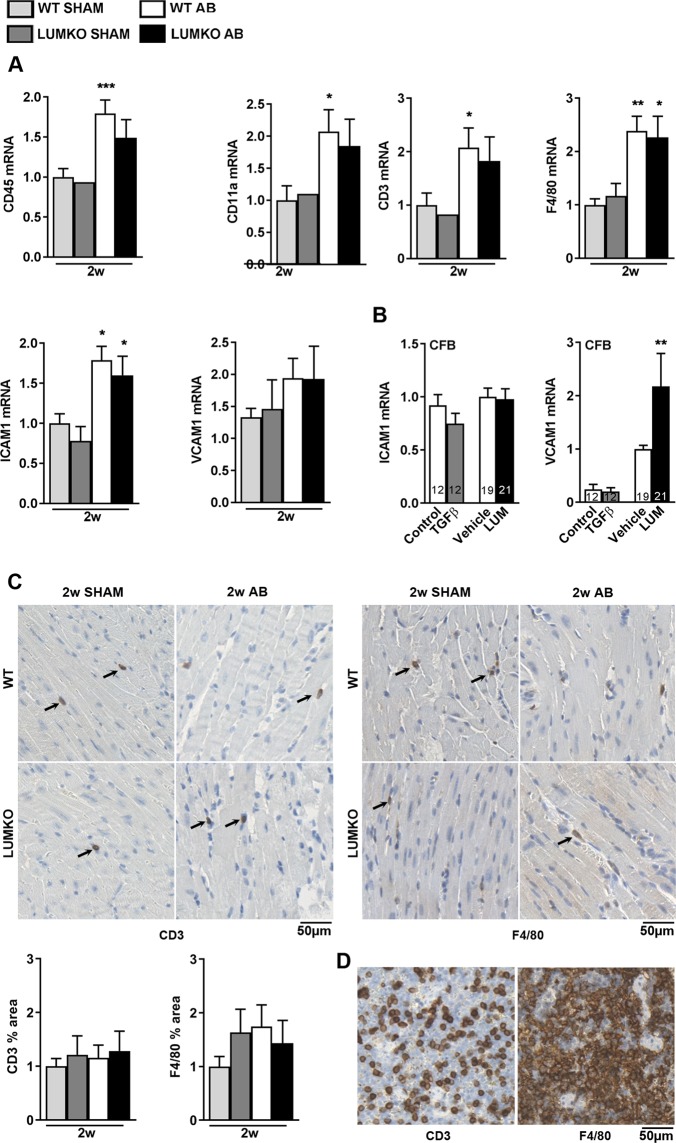
No alteration of immune cell infiltration in LUMKO mice post-AB. (**A**) left ventricular (LV) mRNA expression of immune cell surface markers (leukocytes, CD45 (encoding cluster of differentiation 45) and CD11a (encoding cluster of differentiation 11), T-cells, CD3 (encoding cluster of differentiation 3), and macrophages, F4/80 (encoding adhesion G protein-coupled receptor E1) and immune cell adhesion molecules (ICAM1 and VCAM1, encoding intercellular adhesion molecule-1 and vascular cell adhesion molecule 1, respectively) in LUMKO and WT mice 2w post-SHAM and -AB operations (n SHAM 1–5, n AB 9–10). (**B**) mRNA expression of ICAM1 and VCAM1 in cultured neonatal rat cardiac fibroblasts (n = 3 cell isolations) treated with LUM or vehicle conditioned medium for 24 h. Non-treated cells (control) and cells treated with the pro-fibrotic growth factor transforming growth factor (TGF) β1 served as controls. mRNA expression was normalized to expression of ribosomal protein L32 (RPL32). (**C**) Representative immuno-stained mid-ventricular sections and quantitative measurement of CD3 (T-lymphocyte) and F4/80 (macrophage) infiltration markers in LUMKO and WT 2w post-SHAM and -AB (n WT SHAM = 4, n WT AB = 6, n LUMKO SHAM = 3, n LUMKO AB = 8). Adult WT mouse spleen sections were stained for CD3 and F4/80 as positive controls (**D**). Scale bars 50 µm. Data are presented as mean ± SEM. Differences were tested using one-way ANOVA with Dunn’s post-hoc test vs. WT SHAM (**A**), or an unpaired t-test vs. WT SHAM (A CD45) or vs. Control (**B**), ***p < 0.005; **p < 0.01; *p < 0.05.

To examine whether the exacerbated cardiac phenotype of LUMKO mice post-AB was associated with alterations in cardiac immune cell infiltration, mRNA expression of immune cell surface markers (leukocytes, CD45 (encoding cluster of differentiation 45) and CD11a (encoding cluster of differentiation 11), T-cells, CD3 (encoding cluster of differentiation 3), and macrophages, F4/80 (encoding adhesion G protein-coupled receptor E1) was measured in hearts of LUMKO and WT mice 2w post-AB, and expression of immune cell adhesion molecules (ICAM1 and VCAM1, encoding intercellular adhesion molecule-1 and vascular cell adhesion molecule 1, respectively) was measured in neonatal cardiac fibroblasts treated with LUM. However, we found no differences in CD45 (Fig. 5A), CD11a (Fig. 5A), CD3 (Fig. 5A), F4/80 (Fig. 5A), ICAM1 (Fig. 5A) or VCAM1 (Fig. 5A) in LUMKO hearts vs. WT 2w post-AB, or in ICAM (Fig. 5B) or VCAM (Fig. 5B) in LUM- vs. vehicle-treated cardiac fibroblasts in culture. We also stained mid-ventricular sections for CD3 and F4/80 to detect potential differences in T-lymphocyte and macrophage infiltration between LUMKO AB and WT AB, and there were no significant differences between groups (Fig. 5C,D). Thus, our results indicated that LUM did not affect immune cell infiltration to the pressure-overloaded heart *in vivo*.

### Identification of novel lumican-dependent molecular mechanisms in the pressure-overloaded heart

**Figure 6:**
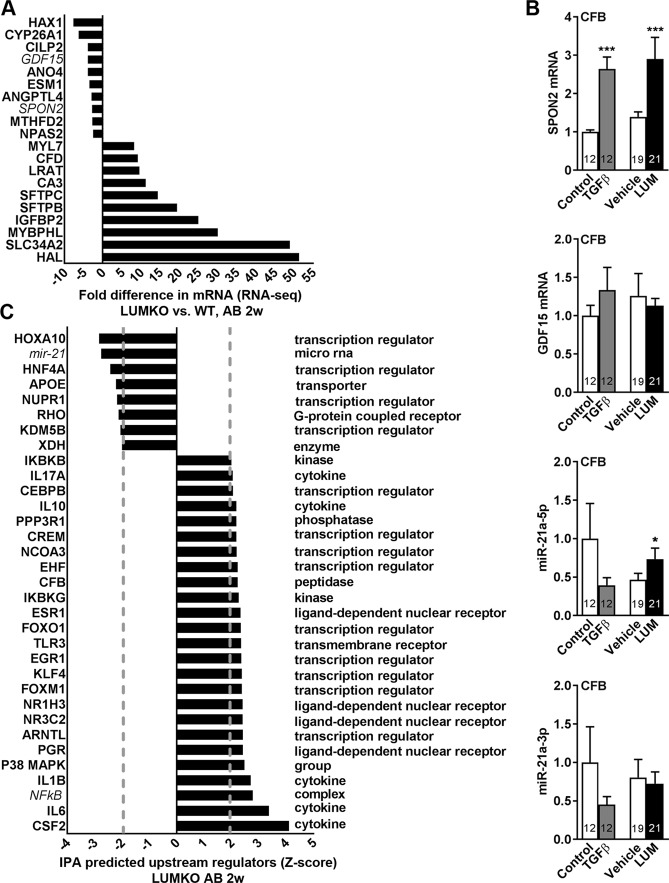
RNA-sequencing revealed novel LUM-dependent molecular mechanisms associated with cardiac remodeling. Lumican knock-out (LUMKO) and wild-type (WT) mice were subjected to aortic banding (AB) and left ventricles (LV) harvested at 2 weeks (w) post-AB for RNA-sequencing of pools of n = 3. 714 differentially expressed (DE) transcripts were identified, i.e. 526 > 1.33-fold up- and 188 < 0.75-fold down-regulated, (p < 0.001), please see Suppl. File. (**A**) Top 10 down-regulated (except LUM itself, which was not expressed) and top 10 up-regulated transcripts in LUMKO vs. WT. (**B**) Cultured cardiac fibroblasts from neonatal rats (n = 3 cell isolations) were treated with LUM or vehicle conditioned medium for 24 h. Non-treated cells (control) and cells treated with the pro-fibrotic growth factor transforming growth factor (TGF) β1 served as controls. mRNA expression of spondin-2 (SPON2) and growth differentiation factor 15 (GDF-15) identified in (**A**); and mRNA expression of miR-21-5p and -3p, respectively, predicted in (**C**). Expression was normalized to expression of ribosomal protein L32 (RPL32) or U6. (**C**) Ingenuity pathway analysis (IPA) prediction of upstream regulators of DE transcripts in LUMKO vs. WT LVs 2w post-AB (Z-score >2 = activated, <2 = inactivated). The data are presented as mean ± SEM. Differences were tested using an unpaired t-test vs. Vehicle or Control, *p < 0.05; ***p < 0.005.

Transcriptional profiling of the 714 DE transcripts revealed from RNA sequencing of LUMKO and WT hearts 2 weeks post-AB was used to identify novel LUM-dependent molecular mechanism in the pressure-overloaded heart. Looking at the 10 most down- (except LUM, which was not detected) and up-regulated genes in LUMKO hearts (Fig. 6A), SPON2, encoding spondin 2, an extracellular matrix molecule with pro-fibrotic, pro-hypertrophic and pro-inflammatory effects^30,31^ was among the ten most downregulated genes. In line with a direct role for LUM in regulating SPON2 expression, LUM treatment upregulated the expression of SPON2 in cardiac fibroblasts (Fig. 6B).

Similarly, the heart failure signature gene GDF15^32,33^ was among the 10 most down-regulated genes in LUMKO hearts post-AB (Fig. 6A). We found no effect on GDF15 expression in cardiac fibroblasts treated with LUM (Fig. 6B), suggesting an indirect role for LUM in regulating GDF15 in the heart or that the cardiomyocyte GDF15 expression predominates over that of fibroblasts. IPA was used to predict activated and inactivated upstream regulators of the 714 DE transcripts in LUMKO vs. WT hearts 2 weeks post-AB (Fig. 6C). Interestingly, a micro-RNA (miR) strongly implicated in fibrosis, including in the heart, miR-21^34–36^, was predicted by IPA among the top inactivated upstream regulators of the DE transcripts in LUMKO hearts (Fig. 5C). In line with this, LUM treatment of cardiac fibroblasts resulted in increased expression of miR-21a-5p (Fig. 5B) whereas miR-21a-3p was unaffected (Fig. 5B). Nuclear factor (NF)-κB, a major pro-inflammatory transcription factor^37^, was among the top predicted activated upstream regulators of the DE transcripts in LUMKO vs. WT hearts 2 weeks post-AB (Fig. 6C), supporting previous findings^27^. Altogether, transcriptional profiling of DE transcripts from RNA-sequencing identified that LUM, directly or indirectly, might be involved in regulating levels of the ECM component spondin 2, GDF15 and the central fibrosis-regulating miR, miR-21, in the heart.

## Discussion

The present study shows that LUM plays a critical role in cardiac remodeling following LV pressure overload. LUMKO mice exhibited increased early mortality upon AB, and surviving LUMKO mice displayed increased LV dilatation, altered hypertrophic growth and increased contractile dysfunction compared to controls. Increased lung weights and dilated atria were consistent with exacerbated congestive heart failure in LUMKO mice compared to WT^38^. Collagen cross-linking and collagen mRNA levels were reduced in LUMKO hearts, and there was a tendency towards reduced collagen protein in LUMKO compared to WT hearts post-AB. Transcriptional profiling revealed 714 DE transcripts in LUMKO hearts vs. WT after AB, with enrichment of cardiotoxicity pathways consistent with the observed cardiac phenotype, and gene ontology and KEGG pathways involved in ECM remodeling and inflammation. We found no differences in cardiac immune cell infiltration post-AB in WT and LUMKO mice. Experiments in cultured cardiac fibroblasts revealed that LUM directly affects mRNA levels of markers of fibroblast-to-myofibroblast transdifferentiation, proliferation, and production of collagens, the collagen cross-linking enzyme LOX, levels of the pro-fibrotic miR-21 and ECM components such as periostin, MMP2, hyaluronan and spondin 2. Transcriptional profiling of the DE transcripts in LUMKO hearts vs. WT after AB, in combination with experiments in cultured cardiac fibroblasts, confirmed that LUM directly regulated mRNA levels of ECM components.

Over the last two decades, SLRPs including LUM have been shown to have a role in ECM remodeling and fibrosis in different cardiovascular diseases^39–41^. LUM is increased in rodent models of ischemic-reperfusion and myocardial infarction. Moreover, LUM is abundant in fibrotic lesions of ischemic and infarcted hearts, suggesting a role for LUM in cardiovascular diseases^39,40^. We have previously shown that LUM mRNA and protein levels are increased in hearts of patients with end-stage dilated cardiomyopathy (DCM) and in hearts of mice after pressure overload^27^, suggesting a role for LUM in cardiac remodeling. Here we tested this hypothesis, subjecting WT and LUMKO mice to AB. Importantly, LUMKO exhibited increased early mortality 1–21 days following AB, indicating that LUM plays a crucial role in the myocardial response to increased stress caused by pressure overload. Moreover, surviving LUMKO mice showed exacerbated LV dilatation, altered hypertrophic growth, and exacerbated contractile dysfunction and heart failure post-AB. Thus, our data suggest that LUM is important for survival following pressure overload with counteracting LV dilatation and cardiac dysfunction in the heart.

The most important *in vivo* phenotype of mice lacking LUM, i.e. increased LV dilatation and exacerbated dysfunction, could be caused by altered properties of the cardiomyocytes and/or of the ECM, of which collagens are vital for tissue integrity. LUM is an ECM keratan sulfate SLRP binding to fibrillar collagens and regulating collagen fibrillogenesis^29^, thus affecting the quality of ECM. It is critical for collagen fibril growth and maintaining the interfibrillar space of collagens, and corroboratively LUMKO mice show loss of connective tissue structural integrity^42,43^. Collagen elasticity and strength prominently relies on cross-linking^44^, proposing that it is not only the amount of collagen, but also cross-linking that determines cardiac chamber remodeling. In the present study, we found that collagen cross-linking was diminished in LUMKO hearts. This was supported by our finding that LUM stimulates the expression of the collagen cross-linking enzyme LOX in cardiac fibroblasts. Thus, reduced collagen cross-linking in LUMKO might lead to breaks in the myocardial collagen matrix and increase side-to-side slippage of collagen fibrils and myocytes, and subsequently lead to increased dilatation. This hypothesis is supported by other findings that a loss of collagen support due to reduced collagen cross-linking might contribute directly to LV dilatation and systolic dysfunction^4^. Further, a decrease in collagen cross-linking parallels LV dilatation in rat models of LV dysfunction and patients with idiopathic DCM^45,46^. Since the collagen matrix of ECM also provides structural integrity for cardiomyocyte shortening and relaxation, it is important for contractile function^47^. Hence, the exacerbated contractile dysfunction of LUMKO hearts might be related to insufficient ECM integrity. Taken together, these data indicate that LUM has a crucial role in maintaining the integrity of cardiac ECM.

During pressure overload, the heart normally responds initially by concentric remodeling and the cardiomyocytes undergo hypertrophic growth^2,47^. If the process is left untreated, the heart might decompensate with resulting left ventricular dilatation and reduced contractility. Here, we demonstrated that wall thickness and cardiomyocyte CSA were reduced in LUMKO compared to WT following pressure overload, demonstrating attenuated concentric hypertrophic growth with resulting LV dilatation. Thus, in the absence of LUM, the myocardium is not able to respond adequately to increased wall stress during pressure overload. LUM has been suggested to control cardiomyocyte growth by regulating the peri-cellular ECM, and LUMKO mice have been shown to have increased cardiomyocyte hypertrophy under physiological conditions without increased cardiac stress^20^. We were not able to measure differences under physiological conditions in our study. This might be explained by age or gender differences between the two studies, although we cannot exclude possible subtle differences in ECM structure and cardiomyocyte function at baseline. Our findings in LUMKO mice during pressure overload of increased heart weight, increased LV dilatation and reduced CSA might be explained by an elongation of cardiomyocytes, i.e. eccentric hypertrophy, but still with an increase in total cardiomyocyte mass. ANP, BNP, MYH7/MYH6 and GDF 15 were all transiently increased 2 weeks after banding both in WT and LUMKO with no difference between the two groups. Although we could expect differences between groups because of the differences in phenotype and HF, these findings might be explained by the attenuated concentric hypertrophic growth and lack of adequate response to stress in LUMKO mice.

Following pressure overload, cardiac fibroblasts normally transdifferentiate into myofibroblasts, characterized by proliferation, contractility (αSMA expression) and increased ECM/collagen production^48–50^, leading to increased stiffness of myocardium and counteracting dilatation. In the present study, LV dilatation in LUMKO mice was accompanied by reduced cardiac expression of COL1A2 and COL3A1, and mRNA levels of the myofibroblast markers αSMA and SM22. We also measured a tendency to reduced levels of αSMA protein in LUMKO AB compared to WT AB at 2 and 12 weeks. Cultured cardiac fibroblasts treated with LUM responded with increased mRNA expression of αSMA and SM22, a proliferation marker, and expression of ECM molecules including COL1A2, COL3A1 and periostin. These data are in line with LUM being involved in the fibrotic process having direct effects on collagen production and maturation i.e. cross-linking, a finding supported by Dupuis *et al*. reporting reduced cardiac collagens in LUMKO mice^20^. In contrast, Chen *et al*. recently reported that lack of LUM exacerbates isoproterenol-induced cardiac fibrosis through up-regulation of the TGF-β and MMP signaling pathways, suggesting that LUM might have a protective role in isoproterenol-induced cardiac fibrosis^51^. In our study we showed a tendency towards increased TGFβ1 expression in CFB stimulated with LUM and we have previously shown that LUM increases the expression of TGFβ1 and phosphorylation of SMAD3 in cardiac fibroblasts *in vitro*^27^, supporting a profibrotic effect of LUM and not antifibrotic as also reported^51^. The reason for the discrepancies between these two studies is not obvious. One explanation might be that Chen *et al*. used another experimental model where stress was induced by pharmacological administration of a drug that might have several effects other than increasing LV pressure, as was obtained mechanically in the present study by AB.

Spondin 2 was identified as a novel downstream target of LUM in cardiac fibroblasts. Increased expression of spondin 2 has been reported during cardiac hypertrophy^31^. Spondin 2 is an ECM molecule with pro-fibrotic, pro-hypertrophic and pro-inflammatory effects^30,31^. Since spondin 2 is known to attenuate maladaptive cardiac remodeling in mice after pressure overload, preventing transition to HF, the severely reduced spondin 2 level in LUMKO mice is likely to represent a mechanism contributing to increased dilatation and reduced hypertrophic growth^31,52^. Another important, novel, and exciting finding of our study is that levels of the fibrosis-associated miR-21^34,35,53^ expressed by cardiac fibroblasts are directly regulated by LUM. This combined with an increase in MMP-2, periostin, HAS2 and PCNA mRNA by LUM in CFB, suggest an important role for this interaction in myocardial remodeling.

Lumican is known to be important for tissue inflammation. It affects immune cell recruitment after corneal injury^24,25^, and activates innate immune responses through binding to the TLR4 adaptor CD14^26^. Its expression in cardiac fibroblasts is up-regulated by mechanical stress, eliciting inflammation, interleukin-1β, and lipopolysaccharide (LPS)^27^, consistent with a role in innate immunity. However, it is not known whether lack of LUM affects inflammation in the pressure overloaded heart. In the present study we were not able to demonstrate differences in immune cell infiltration between WT and LUMKO 2 weeks after AB. However, we have previously shown that recombinant LUM activates NF-κB signaling in cultured cardiac fibroblasts^27^, and in line with this, NF-κB was predicted as an upstream regulator of DE transcripts in LUMKO hearts.

In addition to the myocardium, LUM is expressed and believed to have a function in other organs such as blood vessels, pulmonary and aortic valves, and lung^16,54–56^. LUM has been shown to be produced by vascular smooth muscle cells^57^, hence it might regulate the integrity of blood vessel walls. Moreover, patients with pulmonary fibrosis show increased LUM in the fibrotic lesions^16^. Thus, although not the aim of this particular study, lack of LUM might therefore affect systems and organs also beyond the myocardium that might potentially contribute to the findings of our study.

Our study has some limitations. The high perinatal mortality of LUMKO mice clearly indicates that LUM is important for normal development and is in concordance with previous results^12,20^. However, the cause of increased mortality in LUMKO mice at baseline was beyond the scope of this study. Feeding the LUMKO mice with wet feed after birth increased their survival. The dramatically increased mortality of LUMKO mice reflected in a lower than predicted numbers of live-born pups, combined with substantially increased perioperative mortality resulted in a relatively low number of mice reaching 12 postoperative weeks after AB in our *in vivo* study. Thus, we cannot exclude that type II statistical errors have occurred. Future studies might focus on the more robust heterozygous LUMKO or transgenic LUM mice to obtain better survival in *in vivo* studies. Moreover, conditional knock-out of the LUM gene in fibroblasts or other cell types might be another strategy to reveal the functional role of LUM.

In conclusion, our study reports the novel finding that LUM is important for survival and cardiac remodeling in response to pressure overload. Lack of LUM reduced survival, altered hypertrophic remodeling, increased contractile dysfunction, LV dilatation and lung weight, all pivotal processes in the progression to heart failure. LUMKO mice showed reduced collagen cross-linking likely disrupting the ECM integrity and contributing to the exacerbated cardiac phenotype. LUM regulates the cardiac fibroblast mRNA expression of pro-fibrotic molecules such as collagens, lysyl oxidase and spondin 2, possibly, regulated by miR-21-related pathways.

## Methods and Materials

A detailed description of the methods is provided in Supplementary Material. All the experimental protocols were approved by Institute for Experimental Medical Research (IEMR) at Oslo University Hospital and the methods were carried out in accordance with the relevant guidelines and regulations at IEMR.

### Animal experiments

Animal experiments were approved by The Norwegian Animal Research Committee (protocol IDs 4531 and 11669), and conformed to the Guide for the Care and Use of Laboratory Animals (National Institute of Health (NIH, MD). Aortic banding (AB) or sham operations followed by echocardiography were performed on Adult (8–9 weeks old) female wild-type (WT) and LUMKO mice as previously described^58,59^. Mice were sacrificed by cervical dislocation 2 and 12 weeks post-AB.

### Histology and quantification

Wheat Germ Agglutinin (WGA) staining was performed to measure cardiomyocyte cross sectional area (CSA). Picrosirius Red staining was performed to measure fibrosis and collagen cross-linking using bright field and polarized light, respectively^9^.

### Immunostaining

Immunostaining was performed on mid-ventricular sections to measure T-lymphocyte and macrophage infiltration, respectively using antibody against CD3 (#ab 16669) and F4/80 (#ab 6640) (Abcam, Cambridge, United Kingdom). Detection kits RMR622 (for CD3) and RT517 (for F4/80) (BioCare Medical, Pacheco, CA) were used for detecting positive cells, in which HRP-conjugated secondary antibodies were reacted with DAB as the chromogen. Nuclei were counterstained with hematoxylin.

### HEK293 cell culture and transfection

Human endothelial kidney (HEK) 293 cells were cultured as described^8,10,60,61^. Cells were transfected with a pcDNA 3.1 vector (4 µg) using Lipofectamine 2000 (Invitrogen, Paisley, UK) encoding human LUM (NP_002336.1), LUM with a C-terminal His tag (LUM-His) or non-glycosylated LUMΔGAG (custom made by GenScript Corporation, NJ). Cells and medium were harvested after 48 h. Conditioned medium was used for treatment of rat cardiac fibroblast cultures (CFB)^8,61^. Experiments were performed in three separate cell culture isolations.

### Proliferation assay

Cell proliferation was assessed using the CyQUANT Proliferation Assay Kit (C7026, Thermo Scientific) according to the manufacturer’s instructions (see Supplemental Material).

### Gene expression analysis

Total RNA was extracted, cDNA synthesized and Fast Real Time PCR System or dd-PCR was performed. RNAseq was performed as described^62,63^ on total RNA extracted from LV tissue using Trizol (Thermo Scientific).

### Protein analysis

Immunoblotting was performed on protein lysates from cells and LV tissues as described^8,10,61^. Enzymatic deglycosylation of LUM was performed using PNGaseF (1 µl/1 hr/37 °C), as described^27^.

### Statistics

Data are expressed as mean ± standard error mean (SEM). Statistical analysis was performed using the GraphPad software (Prism 7) and Sigma Plot 11 (Systat Software Inc., San Jose, CA). P < 0.05 was considered as statistically significant. RNA sequencing expression data were analyzed with Bayesian comparison of normalized reads, with p < 0.001 considered significant^62,63^. For pathway and GO analyses, Benjamini-Hochberg (B-H) false discovery rate (FDR) < 0.05 was considered significant. The use of parametric or non-parametric tests was based on results from analyses of distributions. Statistical significance was determined using Student’s t-test and one-way and two-way ANOVA followed by Bonferroni post hoc tests for normally distributed data or Mann–Whitney Rank Sum Test. Pearson’s test was used for correlation analyses. Survival rates were calculated using Log-rank (Mantel–Cox) test.

## Supplementary information

Supplementary info

Dataset 1

## Data Availability

The datasets generated during and/or analyzed during the current study are available from the corresponding author on reasonable request.
