# Supplementary material for: The extracellular matrix proteoglycan lumican improves survival and counteracts cardiac dilatation and failure in mice subjected to pressure overload
Source: Sci Rep. 2019 Jun 24;9:9206. doi: 10.1038/s41598-019-45651-9 (PMC6591256; doi:10.1038/s41598-019-45651-9)
Supplement: Supplementary file 1 — Supplementary info [file 41598_2019_45651_MOESM1_ESM.pdf]

The extracellular matrix proteoglycan lumican improves survival and counteracts cardiac dilatation and failure in mice subjected to pressure overload

Naiyereh Mohammadzadeh MS[1,2], Ida G. Lunde PhD[1,2,3,4], Kine Andenæs MS[1,2], Mari E. Strand PhD[1,2], Jan Magnus Aronsen MD, PhD[1,5], Biljana Skrbic MD, PhD[1,2,6], Henriette S. Marstein MS[1,2], Caroline Bandlien MD[1,2,6], Ståle Nygård PhD[1,2,7], Joshua Gorham BA[4], Ivar Sjaastad MD, PhD[1,2], Shukti Chakravarti MD, PhD[8], Geir Christensen MD, PhD[1,2], Kristin V. T. Engebretsen MD, PhD[1,2,9], Theis Tønnessen MD, PhD[1,2,6,#] .

[1] Institute for Experimental Medical Research, Oslo University Hospital and University of Oslo, Norway.

[2] KG Jebsen Cardiac Research Center and Center for Heart Failure Research, University of Oslo, Norway.

[3] Center for Molecular Medicine Norway, Oslo University Hospital and University of Oslo, Norway.

[4] Department of Genetics, Harvard Medical School, Boston, MA, USA.

[5] Bjørknes College, Oslo, Norway.

[6] Department of Cardiothoracic Surgery, Oslo University Hospital, Norway.

[7] Department of Informatics, University of Oslo, Norway.

[8] Department of Medicine, Johns Hopkins University, Baltimore, MD, USA.

Current: Department of Ophthalmology and Pathology, NYU Langone Health, NY, NY10011

[9] Department of Surgery, Vestre Viken Hospital, Drammen, Norway.

# Corresponding author: Professor Theis Tønnessen, Institute for Experimental Medical Research and Department of Cardiothoracic Surgery, Oslo University Hospital, Kirkeveien 166, 0407 Oslo, Norway.

E-mail: theis.tonnessen@medisin.uio.no

Key words: Small leucine-rich proteoglycan, cardiac remodeling, aortic banding, fibrosis, collagen cross-linking

Declaration of interest: None

SUPPLEMENTAL MATERIAL

Detailed Methods

All the experimental protocols were approved by Institute for Experimental Medical Research (IEMR) at Oslo University Hospital and the methods were carried out in accordance with the relevant guidelines and regulations at IEMR.

Animal experiments

Animal experiments were approved by The Norwegian Animal Research Committee (protocol IDs 4531 and 11669), and conformed to the Guide for the Care and Use of Laboratory Animals (National Institute of Health (NIH, MD). The LUMKO mice used in this study were received from S. Chakravarti and were bred onto C57BL/6J background [1]. After back-crossing 5 times, the LUMKO C57BL/6J background was confirmed by speed congenics at Charles River Laboratories (Wilmington, MA) to have 99, 87 % C57BL6/J background. Intercrosses of heterozygous LUMKO were used to evaluate genotype distribution of pups at weaning age (3-4 weeks of age). To try to increase the distribution of KO pups lumican KO males were crossed with heterozygous LUMKO females. Homozygous LUMKO females are not recommended for breeding (personal communication Chakravarti). To optimize mouse breeding the male was given a week to make his new cage territory scent marked before two-tree heterozygous LUMKO mice were introduced. Noise and traffic were kept to a minimum and the mice were given nesting material and environmental enrichment. The mice were kept on a regular light/dark circle. Disturbance of gestating females and new litters were kept to the least possible. Genotyping was performed on ear biopsies [1]. Adult (8-9 weeks old) female wild-type (WT) and LUMKO mice were subjected to banding of the ascending aorta (AB) by an experienced researcher blinded to genotype, as previously described [2]. SHAM operation consisted of the same procedure without tightening of the ligature. The first mice that were operated were used for characterization of phenotype until 12 weeks post AB. The LUMKO mice showed a distinct and significant phenotype from 2 weeks post AB and we therefore chose 2 weeks post AB as a time-point for further analysis. During surgery, mice were incubated and ventilated with a mixture of 98% oxygen and 2% isoflurane on a Mini-Vent ventilator (Harvard Apparatus, Holliston, MA). AB was performed through a left-sided, muscle-saving thoracotomy under a dissecting microscope (Carl Zeiss Microscopy GmbH, Jena, Germany). Mice received pre- and post-operative analgesia by subcutaneous injection of 0.02 ml (0.3 mg/ml) buprenorphine. Animals with sufficient degree of aortic constriction (maximal flow velocity (Vmax) of 3-4 m/s over the stenosis) 24h post-AB were included. The mean gradients were comparable in both banded groups (3.65±0.08 m/s and 3.71±0.11 m/s in WT and KO, respectively). Echocardiography was performed by an experienced researcher blinded to genotype before AB (baseline), and 1, 2, 4, 6 and 10 weeks after AB, on mice breathing a mixture of 1.75% isoflurane and 98.25% O_2 on a mask, using the VEVO 2100 system (VisualSonics, Toronto, Canada). We are missing echocardiographic results from those LUMKO mice in week 12 that were observed to be in end-stage heart failure with pulmonary edema and about to die. For animal welfare it was necessary with abrupt sacrifice without waiting for echocardiography. However, their hearts were harvested to be used for histology and molecular biology. Mice were anesthetized with 5% isoflurane and sacrificed by cervical dislocation 2 and 12 weeks post-AB. Heart and lungs were rapidly excised, rinsed in PBS and blotted dry. Heart and lung weights were weighed, and normalized to body weight. Hearts were cut with a razor blade at the mid-ventricular plane. The basal bi-ventricular part was used for histology, while from the apical part, the LV was dissected, snap-frozen in liquid nitrogen and stored at -70° for RNA or protein analyses.

HEK293 cell culture and transfection

Human endothelial kidney (HEK) 293 cells were cultured as described [3-6]. Cells were transfected with a pcDNA 3.1 vector (4 µg) using Lipofectamine 2000 (Invitrogen, Paisley, UK) encoding human LUM (NP_002336.1), LUM with a C-terminal His tag (LUM-His) or LUMΔGAG (custom made by GenScript Corporation, NJ). LUMΔGAG was made by site-directed mutagenesis of Asn (N) for Ala (A) residues of GAG N-glycosylation attachment sites in LUM, i.e. N88A, N127A, N160A, N252A. Non-transfection or transfection with empty pcDNA3.1 vector (vehicle) served as controls. Cells and medium were harvested after 48h. Conditioned medium for treatment of primary heart cell cultures was cleared by centrifugation at 5000g. Successful transfection of HEK293 cells was confirmed by immunoblot or His ELISA (#L00436, GenScript) of conditioned medium. For immunoblot analyses, cell lysates were harvested, and medium concentrated using Amicon Ultra-4 centrifugal filters (3 kDa cut-off, Merck Millipore, Darmstadt, Germany). Protein was stored at -70°C. Medium and cell lysates from three separate experiments were used.

Cardiac fibroblast culture

Primary rat cardiac fibroblast cultures (CFB) were prepared from 1–3day old Wistar rats (BomTac, Taconic, Skensved, Denmark) essentially as described [4,6]. Following 24h of serum deprivation (1% PS (penicillin/streptomycin (P0781; Sigma)), CFB were treated for 24h with 2 ml conditioned medium (diluted 2:1 in fresh Dulbecco's modified Eagle's medium including 10% FBS (14-701E; Bio-Whittaker/Lonza, Verviers, Belgium) and 1% PS) from HEK293 cells

overexpressing LUM or vehicle, before harvest of RNA. Non-treated cells served as negative controls, and cells treated with transforming growth factor (TGF)β1 (10ng/ml, GF111, Merck Millipore) served as positive controls. Experiments were performed in three separate cell culture isolations. CFB were passaged only once before stimulation with conditioned medium.Fibroblasts were transferred to fibroblast plating medium (Dulbecco's modified Eagle's medium supplemented with penicillin/streptomyocin and FBS) and maintained in culture for up to 1 week, before being passaged and seeded onto six-well culture plates at a density of 1.89×10^5/ml.

Proliferation assay
Cell proliferation was assessed using the CyQUANT Proliferation Assay Kit (C7026, Thermo Scientific) according to the manufacturer's instructions. Briefly, neonatal CFBs were seeded in 12-well plates at a density of 50000 cells/well, serum-deprived and treated for 24h with conditioned medium containing LUM or vehicle. Cells were detached with trypsin-EDTA (T3925, Sigma), the cell suspension centrifuged for 5 min at 1500 rpm, the supernatant removed and the cell pellet frozen at -80°C for 96h. Subsequently, the cell pellet was suspended in 500 µl CyQuant solution to obtain a cell density within the linear range of the assay (50-50000 cells per 200 µl sample). The fluorescence intensity was measured with excitation at 485 nm and emission at 535 nm.

Gene expression analysis
Gene expression analyses were performed by qRT-PCR, dd-PCR and RNA sequencing (RNAseq) on RNA extracted from cells and LV tissues. For qRT-PCR and dd-PCR, total RNA including microRNA was extracted using the miRNeasy Mini Kit (#217004, Qiagen, Hilden, Germany) according to the manufacturer's protocol. RNA concentration was measured using the Nanodrop ND-1000 Spectrophotometer (Thermo Scientific, Waltham, MA). cDNA was made using iScript cDNA Synthesis Kit (BioRad Laboratories, Inc., Hercules, CA) according to the manufacturer's protocol. Gene expression levels were measured using pre-designed TaqMan assays (Table S7) by 7900HT Fast Real Time PCR System (Applied Biosystems, Foster city, CA) or dd-PCR (Q x200™ Droplet digital PCR system). Results were analyzed using the Sequence Detection System (SDS) 2.3 software (Applied Biosystems). Housekeeping genes RPL32, RPL4 or U6 were used for normalization of gene expression (Fig. S4). RNAseq was performed as described [7 8] on total RNA extracted from LV tissue using Trizol (Thermo Scientific). RNA concentration and quality was measured on Tapestation 2200 (Agilent Technologies, Santa Clara, CA) with RIN >7 accepted. LV libraries were constructed from 3 µg of RNA pooled from the LV of three LUMKO and three WT mice 2w post-AB, for cDNA synthesis using random hexamer priming, without fragmentation or duplex-specific nuclease digestion [9], followed by Nextera XT DNA sample preparation (Illumina, San Diego, CA) according to protocol, including tagmentation and PCR amplification. Libraries were quantified and diluted to 20 nM cDNA and run on the Illumina HighSeq 2500. Data were processed as previously described [9], normalized to total number of reads per sample and expressed as number of fragments per kilobase of exon per million fragments mapped (FPKM). FPKM <2 was considered not expressed. Reads were aligned to the mouse genome (mm10) with a total of 31920 transcripts using Tophat (version 2.0). Cut-off filters used for differentially expressed (DE) transcripts were fold change >1.33 and <0.75 with p<0.001. Ingenuity pathway analysis (IPA, Qiagen), KEGG Pathways and AmiGO gene ontology (GO) analyses were performed on DE transcripts. For IPA predicted upstream regulators (USR), Z-score cut-offs used were ±2 (>2 predicted activation, <-2 predicted inhibition).

Protein analysis
Protein analyses were performed on protein lysates from cells and LV tissues by immunoblotting. LV tissue was homogenized using a Tissue Lyser II (Qiagen) in a 1X PBS-based lysis buffer containing 1% Triton X-100 (Sigma-Aldrich, St. Louis, MO), 0.1% Tween-20 (Sigma-Aldrich), 0.1% sodium dodecyl sulfate (SDS), protease inhibitors (Complete EDTA-free tablets, Roche Diagnostics, Oslo, Norway) and phosphatase inhibitors (PhosStop, Roche Diagnostics), as described [4-6]. Cell lysates were harvested using the same lysis buffer. All samples were spun at 20 000 g for 10 min at 4 °C and the supernatant stored at -80 °C. Protein concentrations were measured using Micro BCA kit (Pierce, #23235; Thermo Scientific). Enzymatic deglycosylation of LUM was performed using PNGaseF (1 µl/1 hr/37˚C), as described [10]. SDS-PAGE was performed using Criterion, 4–15% Tris-HCL gels and proteins transferred to PVDF membranes using the Trans-Blot Turbo Transfer System according to protocol (BioRad). Membranes were blocked in non-fat dry milk (#70166, Sigma-Aldrich), casein (#11921681001, Sigma-Aldrich) or Bovine Albumin (#805095, BioRad) prior to incubation with primary and secondary antibodies. Blots were developed using ECL Prime (GE HealthCare, UK) in the Las-4000 (Fujifilm, Tokyo, Japan) and reprobed after stripping using the Western blot stripping buffer (210591, Thermo Scientific). Processing and quantification were performed using Adobe Photoshop CS5 and ImageQuant TL v.2003.03 and the signal normalized to the loading controls vinculin or Coomassie.

Wheat Germ Agglutinin (WGA) staining and quantification
4 µm mid ventricular sections obtained from all 4 groups (WT SHAM, WT AB, LUMKO SHAM and LUMKO AB) were stained with Wheat Germ Agglutinin (WGA) to examine the average size of cardiomyocyte cross-sectional area (CSA). The histological sections were deparaffinized, hydrated to dH_2O (5min in each: Xylene, Xylene, 100% alcohol, 96% alcohol, 80% alcohol, 70% alcohol and dH_2O). The sections then were rinsed in PBS and boiled for 20min in citrate

buffer PH6 (98^0C) (2.1 g citrate acid monohydrate to 1 L dH2O. Adjust pH to 6.0 by NaOH). Then, sections were cooled down at RT for 20min, rinsed with PBS and incubated with WGA 1:200 in PBS over night at 4^0C. After 24h incubation, sections were rinsed in PBS 1 minx3 and mounted under a coverslip with ProLong™ Gold Antifade Mountant with DAPI (#P36931, Thermo Scientific, Waltham, MA). ImageJ was used to preprocess and threshold the WGA staining to best visualize clear compartments corresponding to cardiomyocytes. One image (i.e. one mid-ventricular section) was analyzed per mouse. The "analyze particles" plugin was used to generate regions of interests, ROIs, of putative cardiomyocytes. Inclusion criteria were defined with ROI size and circularity (a measure of how close to a perfect circle a ROI is). ROI size was set between 90-5000 μm^2, meaning that almost all the ROIs were selected except nuclei which were less than $90\mu m^2$. To exclude longitudinal and strange connected ROIs, we defined a circularity range. With circularity criteria, you only select transverse ROIs (i.e. transverse cross-sectional areas). We have used circularity between 0.6-1.

Picrosirius Red (PSR) staining and quantification
4 µm mid ventricular sections obtained from all 4 groups (WT SHAM, WT AB, LUMKO HAM and LUMKO AB) were stained with Picrosirius Red for the quantification of collagen fibrils. The histological sections were deparaffinized and hydrated to dH_2O (5min in each: Xylene, Xylene, 100% alcohol, 96% alcohol, 80% alcohol, 70% alcohol and dH_2O). The sections then were incubated in solution A (Phosphomolybdic acid hydrate & water) for 2min, and rinsed in dH_2O. Following, the sections incubated in F3BA solution B (2, 4, 6-Trinitrophenol, water & direct Red 80) for 60min, and in hydrochloride solution C (Hydrogen chloride & water) for 2min and in 70% ethanol (45sec). The sections were then dehydrated through alcohol, cleared in xylene and mounted under a cover slip. Fibrosis and cross-linking quantification of PSR stained heart tissue sections was done in ImageJ, using the color threshold plugin to determine the red area corresponding to fibrillar collagens and orange/green area corresponding to cross-linked collagens.

Immunostaining
Immunostaining for CD3 and F4/80 was performed on 4 µm thick paraffin-embedded mid ventricular sections. Briefly, sections were pretreated with Dako Target Retrieval Solution (Tris-EDTA) (pH 9 for CD3, pH 6 for F4/80), blocked with Dako Peroxidase Blocking Reagent for 5 minutes at room temperature (RT), incubated with primary antibodies (anti-CD3 [SP7] (ab16669) 1:100, or anti-F4/80 [CI:A3-1] (ab6640) 1:200 (Abcam, Cambridge, United Kingdom)) for 60 minutes at RT, and treated with HRP-conjugated secondary antibodies and DAB reagent according to manufacturers instruction in the employed kits (RMR622 for CD3, and RT517 for F4/80 (BioCare Medical, Pacheco, CA)). Nuclei were counterstained with hematoxylin. Whole sections were imaged on an automated slide scanner system (Axio Scan Z1, Carl Zeiss Microscopy, Munich, Germany) and quantified in ImageJ. Briefly, images of DAB and hematoxylin positive signals were extracted using the Color Deconvolution plugin [reference here: https://www.ncbi.nlm.nih.gov/pubmed/11531144], and positive pixels were quantified by applying a fixed threshold for each stain and measuring the result.

Antibodies for protein analysis
Primary antibodies used were: anti-Lumican (#AF2745, #A2846, R&D systems, Minneapolis, MN), anti-vinculin (V9131, Sigma-Aldrich, St. Louis, MO), anti-αSMA (A5228, Sigma-Aldrich, St. Louis, MO). Membranes were developed using ECL Prime (Amersham/GEHealthCare, UK) in the Las-4000 (Fujifilm, Tokyo, Japan), followed by stripping with Western blot stripping buffer (210591, Thermo Scientific) and reprobing. Images were quantified and processed using ImageQuant TL v.2003.03 and Photoshop CS5.

Supplemental Figures and Figure Legends

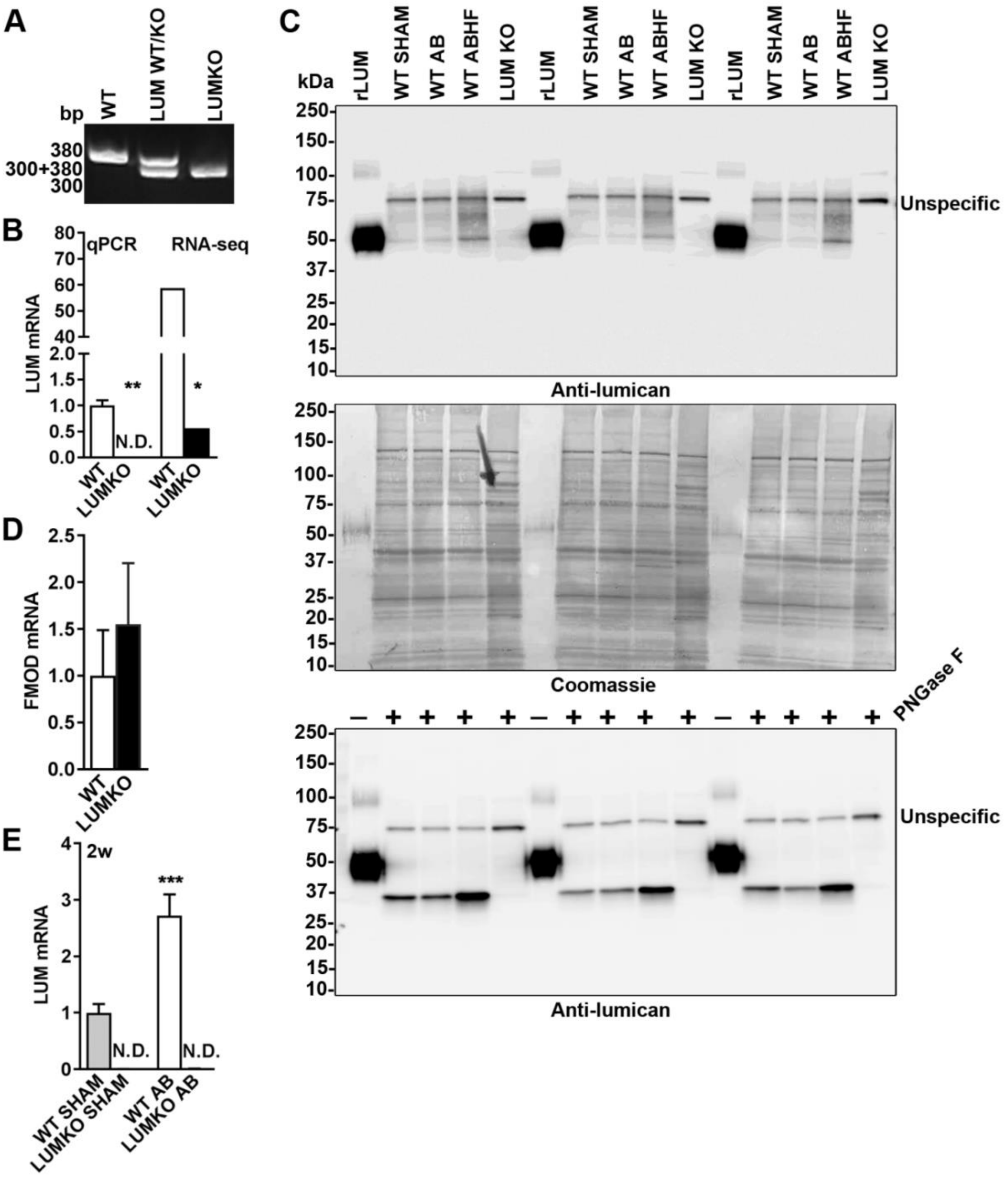

Figure S1. LUMKO mice show no expression of LUM mRNA or protein in the heart. This is the original blot and set up. We have enhanced the contrast slightly and uniformly to the whole blot. (A) Representative genotyping gel of DNA from ear biopsies of WT, heterozygous lumican knock-out (WT/KO) and homozygous lumican knock-out (LUMKO) mice, showing the expected WT band at 380 bp and KO at 300 bp. (B) Relative LUM mRNA in the LV of untreated, adult LUMKO and WT mice measured by qPCR (n=6 per group) and by RNA-sequencing (pools of n=3, 2 weeks post-aortic banding (AB), fragments per kilobase of exon per million reads (FPKM)). N.D. = not detected. (C) Immunoblotting (n=3) showing LUM as a 50-75 kDa proteoglycan in the left ventricle (LV) of LUMKO and WT mice post-AB (with or without heart failure (HF) or SHAM-operation), in a previously published cohort of WT mice [10]. Recombinant LUM (rLUM) was used as control. LUM is shown as a 37 kDa core protein after de-glycosylation with PNGase F, an enzyme which separates all the glycosylated chains from the core protein. (D) Relative FMOD mRNA in the LV of adult LUMKO and WT mice (qPCR, n=6 per group). (E) Relative LUM mRNA in the LV of LUMKO and WT mice 2 weeks post-SHAM and-AB operations. qPCR expressions was normalized to expression of ribosomal protein L32 (RPL32). Data presented as mean ± SEM. Differences were tested using an unpaired t-test vs. WT, $**P<0.05$; $*p<0.05$ (B (qPCR) and D), or vs. WT SHAM, $***P<0.0001$ (E).

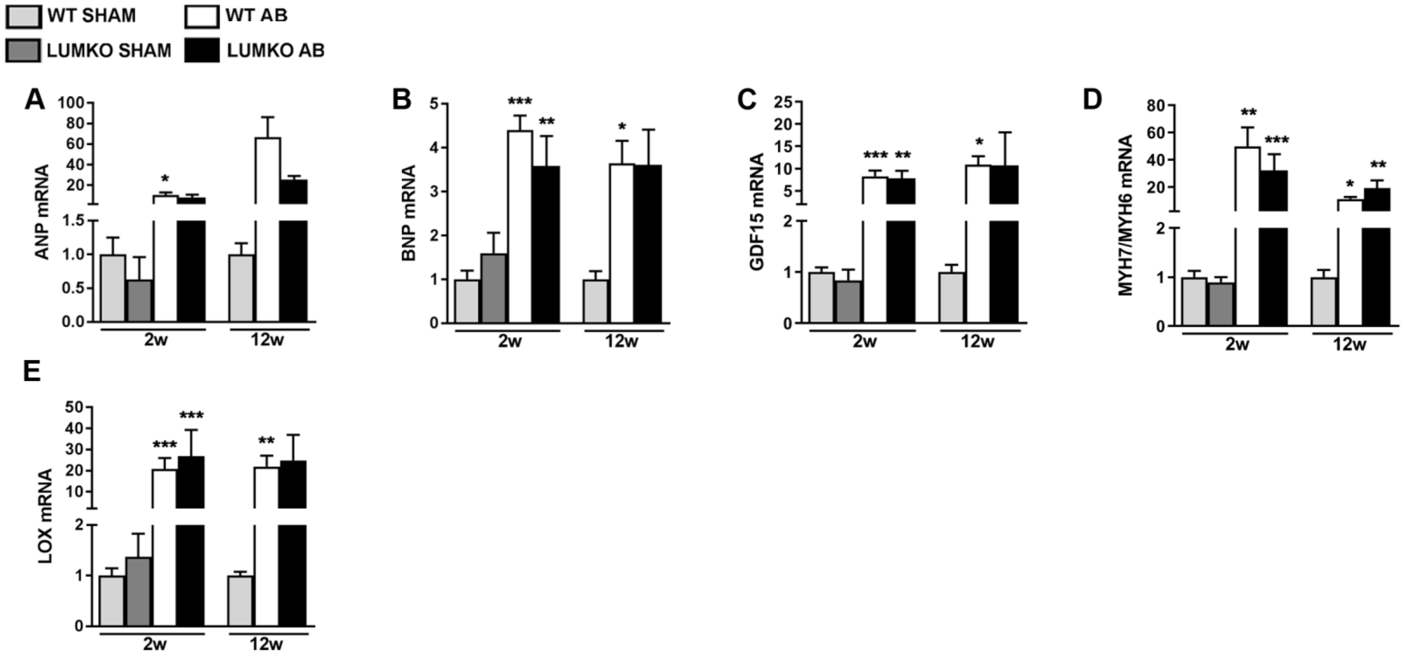

Figure S2. Gene expression of cardiac markers of heart failure and hypertrophy, and collagen cross-linking enzyme in lumican knock-out (LUMKO) and WT mice 2 and 12 weeks post-SHAM and -AB. (A-E) Relative mRNA of atrial natriuretic peptide (ANP), brain natriuretic peptide (BNP), growth differentiation factor 15 (GDF15), myosin heavy chain β/α ratio (MYH7/MYH6) and collagen cross-linking enzyme LOX in the LV of LUMKO and WT mice 2w and 12w post-SHAM and -AB (n SHAM 4-14, n AB 3-22). Expression was normalized to expression of ribosomal protein L32 (RPL32). Data presented as mean ± SEM. Differences were tested using one-way ANOVA with Dunn`s post-hoc test vs. WT SHAM, ***$p<0.005$; **$p\leq0.01$; *$p<0.05$ (A-E).

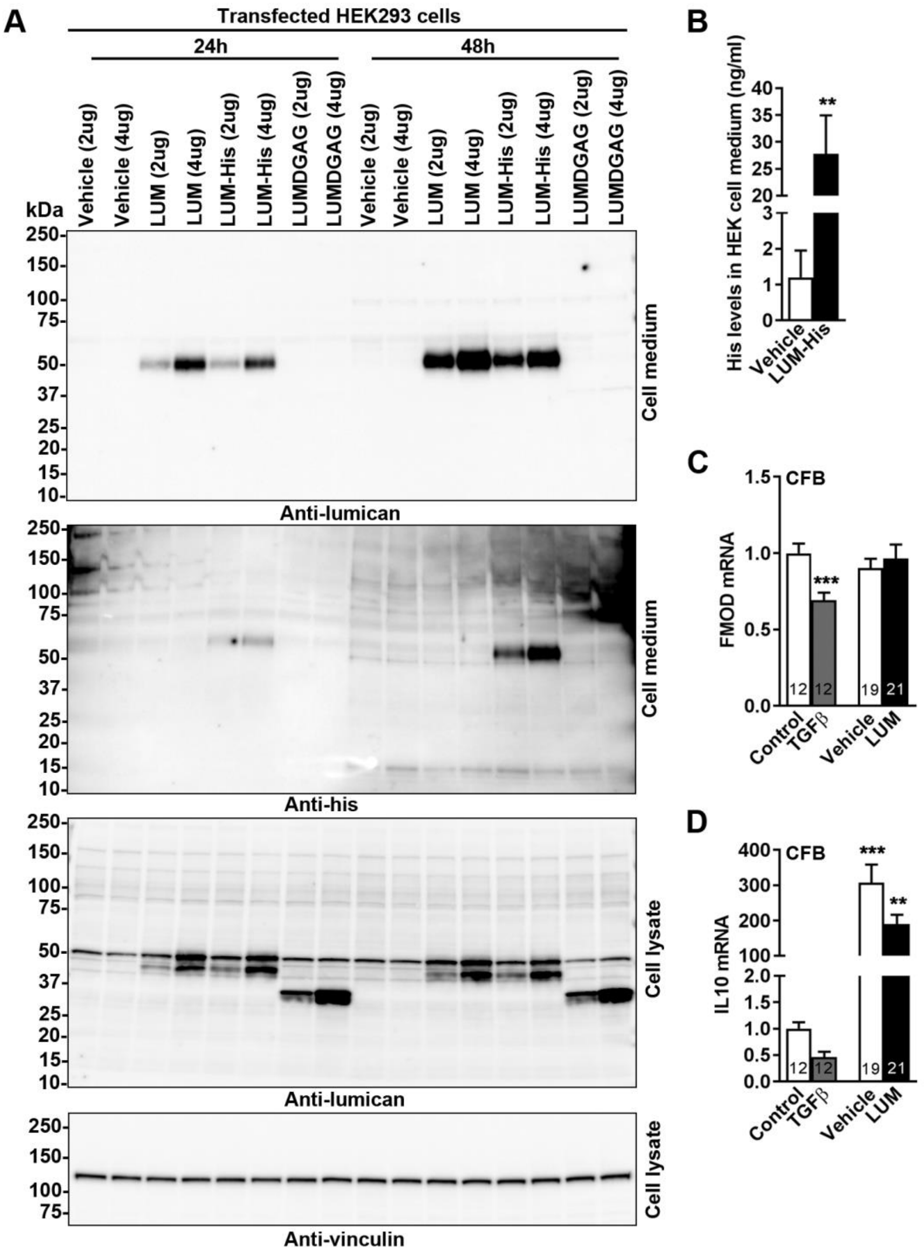

Figure S3. Transfection of human endothelial kidney (HEK) 293 cells with lumican. This is the original blot and set up. We have enhanced the contrast slightly and uniformly to the whole blot. (A) Transfection of HEK 293 cells with 2 and 4 µg of pcDNA 3.1 vectors encoding full length glycosylated LUM (LUM), de-glycosylated LUM (LUMΔGAG) and LUM with a C-terminal His tag (LUM-His) for 24h and 48h. (B) LUM-his levels in HEK293 cell medium measured by His-ELISA. (C and D) Cultured cardiac fibroblasts from neonatal rats (n=3 cell isolations) were treated with LUM or vehicle conditioned medium for 24h. Non-treated cells (control) and cells treated with the pro-fibrotic transforming growth factor (TGF) β1 served as controls. mRNA expression of fibromodulin (FMOD) and interleukin (IL)-10 was normalized to expression of ribosomal protein L32 (RPL32). The data are presented as mean ± SEM. Differences were tested using an unpaired t-test vs. Vehicle, $**p<0.01$ (B), or vs. Control, $***p<0.005$ (C-D).

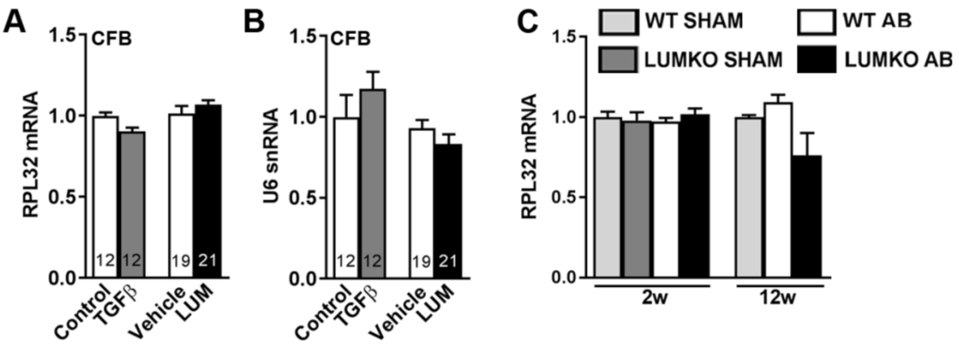

Figure S4. Housekeeping genes used for normalization of qPCR data
(A-C) Gene expression was normalized to ribosomal protein L32 (RPL32) and U6 non-coding small nuclear RNA (U6 snRNA).

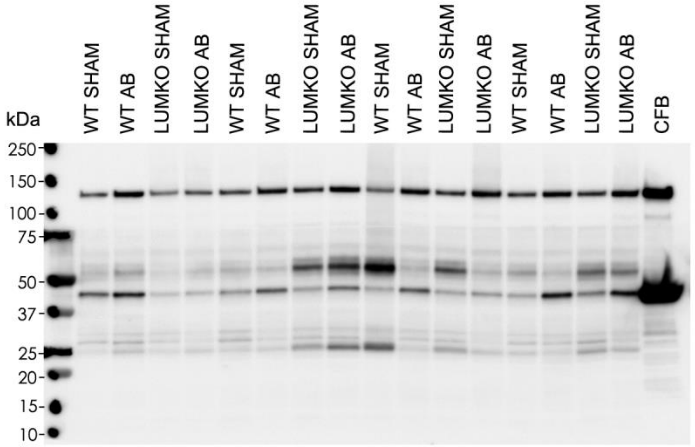

Anti-aSMA

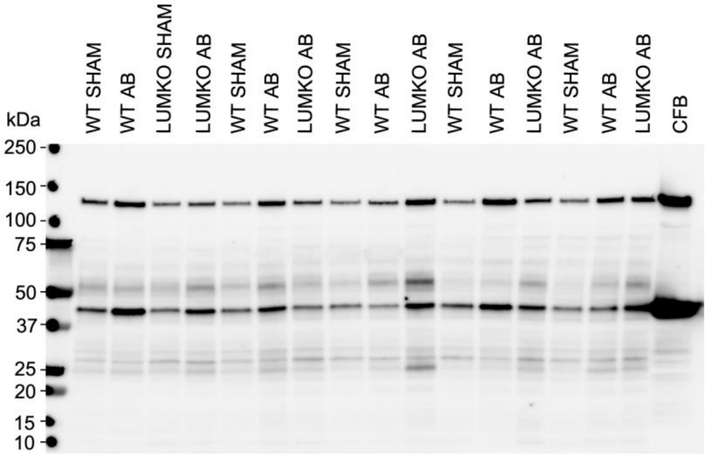

Anti-aSMA

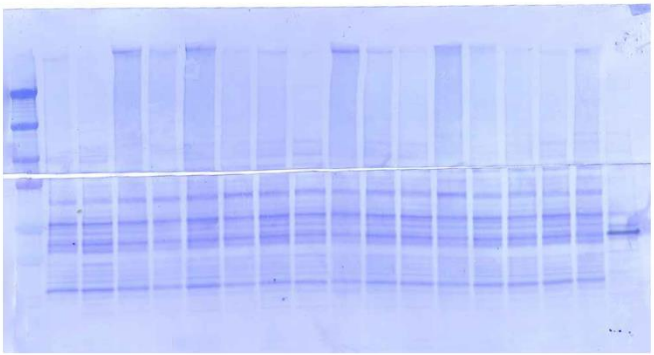

Coomassie

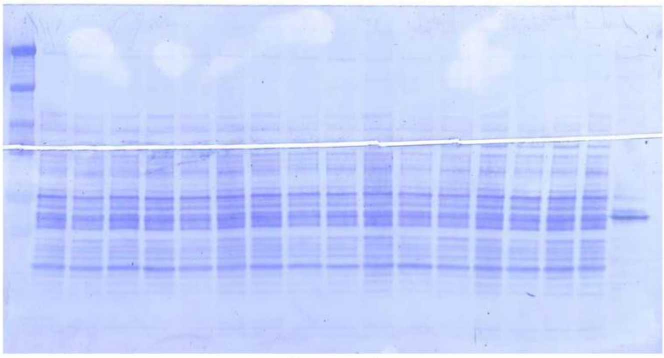

Coomassie

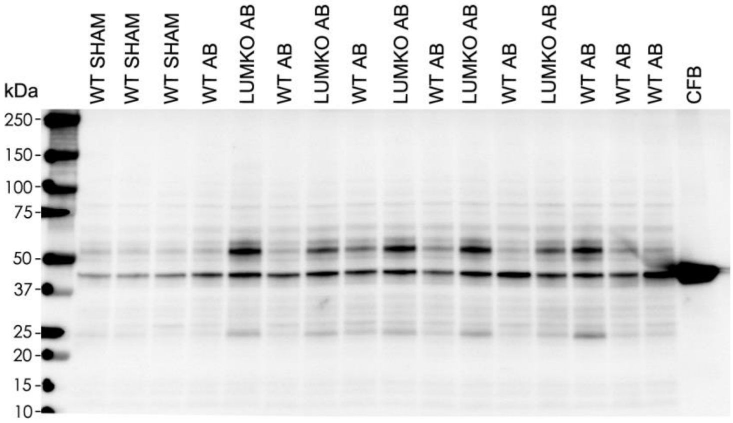

Anti-aSMA

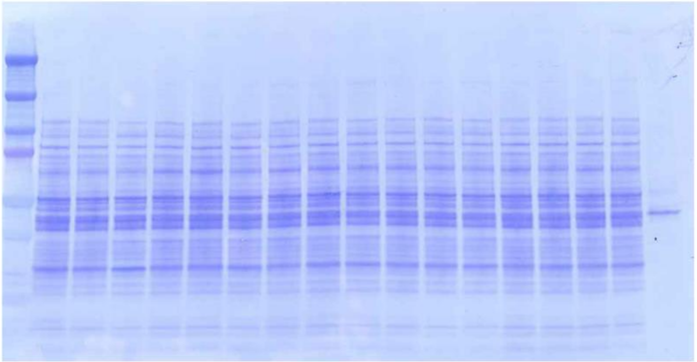

Coomassie

Figure S5. Protein expression of αSMA in LVs of LUMKO and WT mice 2w post-SHAM and-AB operations. Immunoblots of the myofibroblast differentiation marker alpha-smooth muscle actin (αSMA) in left ventricles (LVs) of LUMKO and WT mice 2w post-SHAM and-AB (n WT SHAM = 9, n WT AB = 13, n LUMKO SHAM = 9, n LUMKO AB = 14). Cultured cardiac fibroblasts (CFB) were used as positive control. Coomassie staining was used as loading control.

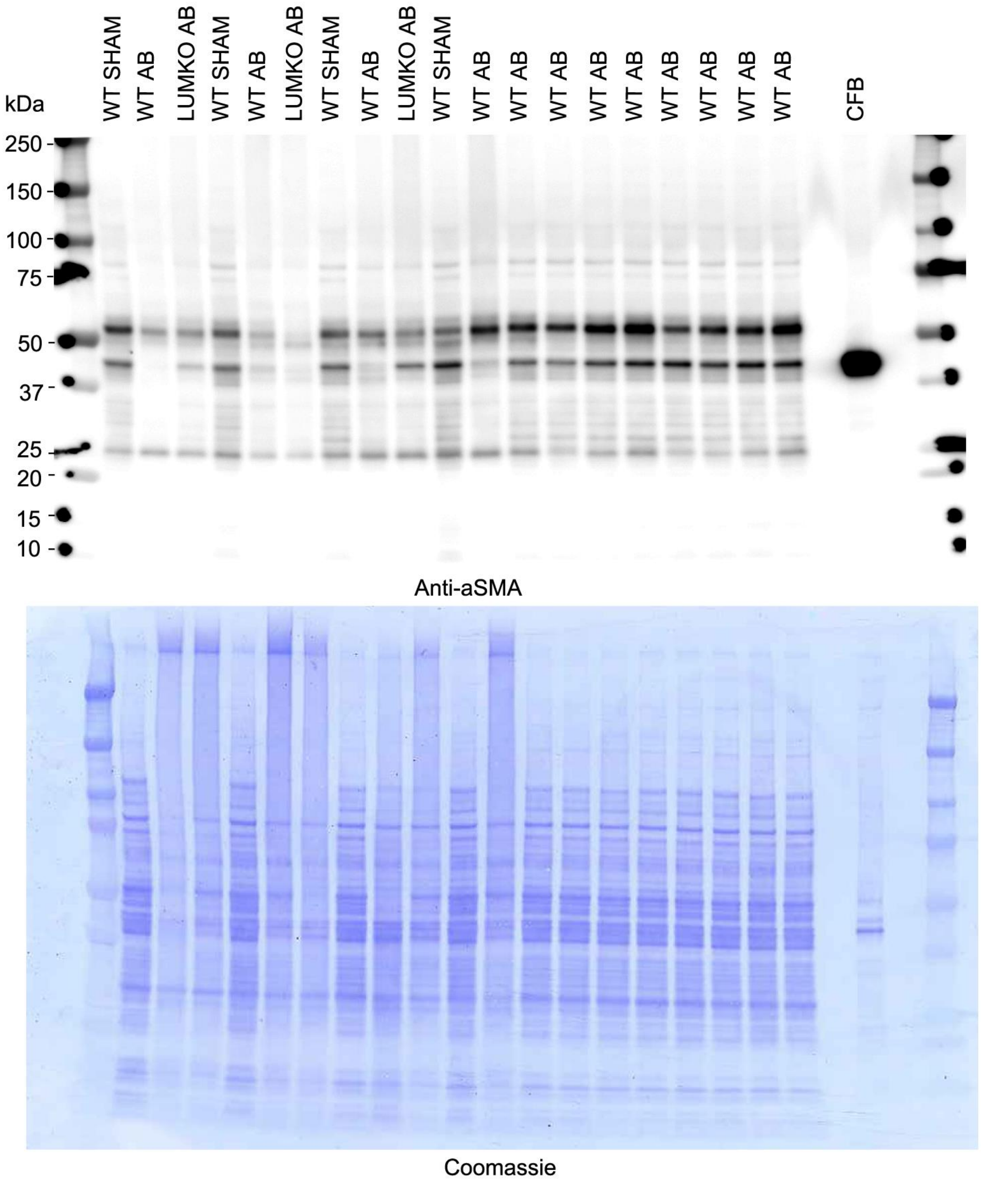

Figure S6. Protein expression of αSMA in LVs of LUMKO and WT mice 12w post-SHAM and-AB operations. Immunoblots of the myofibroblast differentiation marker alpha-smooth muscle actin (αSMA) in left ventricles (LVs) of LUMKO and WT mice 12w post-SHAM and-AB (n WT SHAM = 4, n WT AB = 12, n LUMKO AB = 3). Cultured cardiac fibroblasts (CFB) were used as positive control. Coomassie staining was used as loading control.

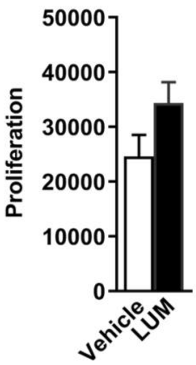

Figure S7. Cell proliferation in cardiac fibroblasts stimulated with vehicle and LUM. Cell proliferation in cardiac fibroblasts stimulated with LUM and vehicle conditioned medium (n=6 per group). Data are shown as mean±SEM.

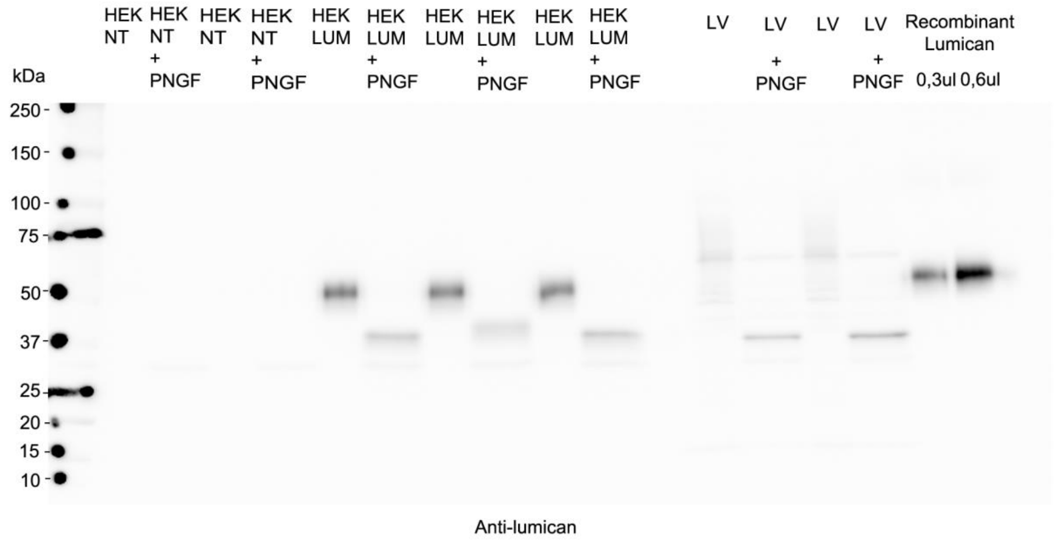

Figure S8. LUM was produced by transfection of human endothelial kidney (HEK)293 cells with human LUM. LUM was secreted into the cell medium as 50 kDa glycosylated proteoglycan. PNGaseF treatment results in a 38 kDa deglycosylated core protein. HEK=HEK-cell medium, NT= non-treated (control), PNGF=PNGase F, LV=left ventricular lysates from a wild-type adult female mice. The second part of this blot using LV samples and recombinant LUM was a second positive control for the experiment. Authors did not see the necessity and relevance of presenting the whole blot; hence we have used the first part (Figure 4). We have enhanced the contrast slightly and uniformly to the whole blot in figure 4.

Supplemental Tables and supporting information

Table S1. Genotype distribution of pups from heterozygous LUMKO intercrosses

WT/WT	WT/KO	KO/KO	Total
**Observed**			
70	158	8	236
30 %	67 %	3%***	100 %
**Expected**			
59	118	59	236
25 %	50 %	25 %	100 %

All live-born pups (n=236) from n=47 heterozygous LUM knock-out (WT/KO) intercrosses were genotyped at weaning age of 3-4 weeks. 8 out of 236 pups were LUMKO (3% vs. expected 25%). Data presented as mean ± SEM. Differences were tested using Chi-squared test vs. expected distribution, $^{***}p<0.005$.

Table S2. Baseline characteristics of LUMKO and WT mice

	WT	LUMKO
n	26	9
IVSd (mm)	0.69 ± 0.01	0.68 ± 0.02
LVIDd (mm)	4.06 ± 0.07	4.03 ± 0.05
PWd (mm)	0.69 ± 0.02	0.67 ± 0.01
IVSs (mm)	0.86 ± 0.02	0.81 ± 0.02
LVIDs (mm)	3.30 ± 0.08	3.36 ± 0.10
PWs (mm)	0.84 ± 0.02	0.80 ± 0.03
FS (%)	18.90 ± 0.93	16.80 ± 1.59
RWT(%)	34.50 ± 1.13	33.70 ± 0.94
LAD (mm)	2.04 ± 0.04	2.16 ± 0.20
n	6	6
BW (g)	23.1 ± 0.6	22.1 ± 0.8
HWBindex (mg/g)	4.71 ± 0.11	4.89 ± 0.12
LWBindex (mg/g)	6.68 ± 0.11	6.80 ± 0.42
ANP (mRNA)	1.00 ± 0.22	1.25 ± 0.22
BNP (mRNA)	1.00 ± 0.18	1.55 ± 0.21
MYH7/MYH6 (mRNA)	1.17±0.29	0.63±0.13
ACTA1 (mRNA)	1.00 ± 0.20	0.87 ± 0.18

Structural and functional properties of LUM knock-out (LUMKO) (n=26) and WT mice (n=9) by echocardiography prior to aortic banding operations (baseline). Organ weights, mRNA expression of heart failure markers and hypertrophic molecules in the LV of adult 9-12 weeks old LUMKO and WT mice (n=6 per group). qPCR data are normalized to RPL32 transcript abundance. WT, wild-type; LUMKO, lumican knock-out; BW, body weight; HWBindex, heart weight/body weight; LWBindex, lung weight/body weight; ANP, atrial natriuretic peptide; BNP, brain natriuretic peptide; MYH7, myosin heavy chain β; MYH6, myosin heavy chain α; ACTA1, actin alpha 1 skeletal muscle; LV, left ventricular; IVSd/s, interventricular septum in diastole/systole; LVIDd/s, LV internal diameter in diastole/systole; PWd/s, posterior LV wall thickness in diastole/systole; FS, fractional shortening; RWT, relative wall thickness; LAD, left atrial diameter. Data are presented as mean ± SEM.

Table S3. Characteristics of LUMKO and WT mice 1-12 weeks post-SHAM or -AB

	1w				2w				4w		6w		10w		12w		
	**WT SHAM**	**WT AB**	**LUM KO SHAM**	**LUM KO AB**	**WT SHAM**	**WT AB**	**LUM KO SHAM**	**LUM KO AB**	**WT AB**	**LUM KO AB**	**WT AB**	**LUM KO AB**	**WT AB**	**LUM KO AB**	**WT SHAM**	**WT AB**	**LUM KO AB**
**n**					14	19	5	13									
**BW (g)**					19.8 ± 0.4	21.3 ± 0.3	19.1 ± 0.5	21.1 ± 0.5									
**n**	14	35	3	17	14	19	5	13	12	3	12	3	12	3	4	10	1
**IVSd (mm)**	0.74 ±0.03	0.98 ±0.02*	0.74 ±0.06	0.88 ±0.03§	0.75 ±0.02	1.01 ±0.03*	0.72 ±0.05	0.89 ±0.03*§	1.07 ± 0.04	0.88 ± 0.50§	1.05 ± 0.03	0.84 ± 0.06§	1.05 ± 0.03	0.88 ± 0.12	0.77 ±0.02	1.03 ±0.03*	1.17
**LVIDd (mm)**	4.00 ±0.11	3.85 ±0.07	4.10 ±0.03	4.35 ±0.11§	4.11 ±0.09	3.94 ±0.09	4.04 ±0.18	4.65 ±0.11*§	3.78 ± 0.19	5.48 ± 0.13§	3.89 ± 0.22	5.99 ± 0.24§	4.25 ± 0.17	5.94 ± 0.29§	4.04 ±0.06	4.38 ±0.20	6.09
**PWd (mm)**	0.74 ±0.04	0.98 ±0.02*	0.71 ±0.08	0.90 ±0.04*	0.74 ±0.02	1.01 ±0.02*	0.71 ±0.05	0.87 ±0.03*§	1.08 ± 0.04	0.85 ± 0.05§	1.04 ± 0.03	0.83 ± 0.07§	1.03 ± 0.04	0.90 ± 0.09	0.78 ±0.03	1.06 ±0.04*	1.10
**IVSs (mm)**	0.94 ±0.05	1.12 ±0.03*	0.96 ±0.10	1.08 ±0.05§	0.92 ±0.03	1.21 ±0.03*	0.95 ±0.06	1.05 ±0.04§	1.26 ± 0.05	0.94 ± 0.07§	1.24 ± 0.03	0.93 ± 0.05§	1.24 ± 0.06	0.94 ± 0.13§	1.02 ±0.03	1.22 ±0.05*	1.24
**LVIDs (mm)**	3.10 ±0.15	3.09 ±0.09	3.13 ±0.12	3.73 ±0.14§	3.26 ±0.12	3.29 ± 0.13	3.24 ± 0.26	4.02 ± 0.16*§	3.11 ± 0.21	5.31 ± 0.11§	3.22 ± 0.26	5.80 ± 0.26§	3.51 ± 0.20	5.75 ± 0.27§	2.94 ±0.13	3.62 ±0.03	5.91
**PWs (mm)**	0.95 ±0.05	1.20 ±0.03*	0.94 ±0.10	1.06 ±0.05§	0.93 ± 0.04	1.26 ± 0.04*	0.90 ± 0.08	1.01 ± 0.05§	1.26 ± 0.06	0.90 ± 0.06§	1.22 ± 0.03	0.88 ± 0.07§	1.19 ± 0.04	0.99 ± 0.12	1.05 ±0.02	1.22 ±0.04*	1.10
**FS (%)**	22.8 ±1.72	19.80 ±1.09	23.60 ±3.38	12.0 ±2.42*§	20.80 ± 1.27	16.70 ± 2.06*	19.70 ± 4.01	14.20 ± 2.49	18.10 ± 2.01	3.05 ± 0.70§	17.80 ± 2.16	3.3 ± 0.7§	17.9 ± 1.7	3.20 ± 0.50§	27.2 ±2.50	18.1 ±2.53	3.0
**RWT (%)**	37.1 ±2.9	51.1 ±1.9*	35.4 ±6.4	39.5 ±4.2§	36.20 ± 1.73	52.10 ± 1.91*	35.40 ± 3.71	35.30 ± 3.14§	58.6 ± 4.2	31.7 ± 2.4§	55.5 ± 3.1	28.1 ± 2.7§	49.4 ± 2.1	30.4 ± 4.5§	38.4 ±0.9	48.80 ±3.09	37.1
**LAD (mm)**	2.00 ±0.10	2.43 ±0.07*	1.91 ±0.24	2.46 ±0.11*	2.03 ± 0.07	2.58 ± 0.08*	1.72 ± 0.16	2.52 ± 0.10*	2.55 ± 0.13	3.18 ± 0.19§	3.03 ± 0.31	3.15 ± 0.24	2.40 ± 0.17	3.42 ± 0.10§	1.80 ±0.07	2.55 ±0.20*	2.99
**n**	9	16	0	11	14	15	5	10	12	3	12	3			4	3	1
**MVE ($mm.s^{-1}$)**	612 ± 32	813 ± 36*	NA	613 ± 31.3§	639 ± 24	763 ± 28*	606 ± 48	706 ± 34	673 ± 114	709 ± 62	674 ± 50	596 ± 78			649±45	594±44	488
**Mdec ($mm.s^{-2}$)**	2670 ± 234	5545 ± 428*	NA	4125 ± 540*	2780 ± 260	4550 ± 300*	2690 ± 530	4700 ± 370*	5250 ± 1180	4150 ± 130	5780 ± 400	3830 ± 790			2619± 243	4476± 1219	3607
**HR**	426 ± 17.8	510 ± 10.3	NA	531 ± 13.6	446 ± 19	492 ± 16	397 ± 31	522 ± 15 *	499 ± 17	544 ± 10	516 ± 10	513 ± 30	559 ± 14	539 ± 33			

Structural and functional, and hemodynamic properties of LUM knock-out (LUMKO) and WT mice by echocardiography 1 to 12 weeks (w) post-SHAM and –AB operations. AB, aorta banding; SHAM, SHAM operated; WT, wild-type; LUMKO, lumican knock-out; BW, body weight; HWBindex, heart weight/body weight; LWBindex, lung weight/body weight; LV, left ventricular; IVSd/s, interventricular septum in diastole/systole; LVIDd/s, LV internal diameter in diastole/systole; PWd/s, posterior LV wall thickness in diastole/systole; FS, fractional shortening; RWT, relative wall thickness; LAD, left atrial diameter; MVE, peak mitral velocity; Mdec, mitral decelaration. Data are presented as mean+SEM. *P ≤ 0.05 AB vs. SHAM. §P ≤ 0.05 KO vs. WT. These data are presented in Fig.2.

Table S4 IPA cardiotoxicity category enrichment of differentially expressed transcripts in hearts of LUMKO 2 weeks post-AB

IPA cardiotoxicity category	IPA Diseases/functions annotation	Over-represented p-value	Multiple testing corrected p-value (FDR)
Cardiac infarction	Myocardial infarction	5.37E-07	3.02E-05
Congenital heart anomaly	Congenital heart block	1.22E-05	0.0003
Cardiac hypertrophy	Hypertrophy of heart	1.68E-05	0.0003
Cardiac dysfunction	Dysfunction of left ventricle	2.85E-04	0.0034
Cardiac necrosis/cell death	Cell death of heart	4.21E-04	0.0045
Cardiac fibrosis	Fibrosis of heart	1.17E-03	0.0073
Cardiac congestive cardiac failure, heart failure	Congestive heart failure	4.66E-03	0.0202
Cardiac arrhythmia	Familial arrhythmia	9.51E-03	0.0340
Cardiac output	Cardiac output	1.33E-02	0.0376
Cardiac enlargement	Enlargement of right ventricle	1.42E-02	0.0376
Cardiac inflammation	Inflammation of heart	1.78E-02	0.0455

IPA cardiotoxicity category enrichment of differentially expressed (DE) cardiac transcripts from lumican (LUM) knock-out (KO) vs. wild-type (WT) mice subjected to two weeks of aortic banding (AB). DE transcripts were identified by RNA sequencing of left ventricular (LV) tissue from LUM KO and WT littermates (n=3), using filters FPKM-fold difference <0.75 and >1.33 with p<0.001, yielding 714 DE transcripts (526 up- and 188 down-regulated). All (11) statistically significant categories (Benjamini-Hochberg (B-H) false discovery rate (FDR) multiple testing correction <0.05) are shown with p-values.

Table S5. Gene ontology enrichment of differentially expressed transcripts in hearts of LUMKO 2 weeks post-AB

Gene ontology (GO) category	Over-represented p-value	Multiple testing corrected p-value (FDR)	#DE transcripts in category
extracellular space	1.70E-19	2.81E-15	54
inflammatory response	1.92E-10	1.58E-06	26
defense response	5.78E-10	3.18E-06	38
response to stimulus	2.91E-09	1.20E-05	142
regulation of multicellular organismal process	4.22E-09	1.39E-05	67
response to stress	8.59E-09	2.36E-05	79
response to external stimulus	3.53E-07	0.0008	46
blood microparticle	4.82E-07	0.001	10
extracellular matrix	1.07E-06	0.002	20
response to chemical	1.78E-06	0.0029	67
multicellular organismal process	3.77E-06	0.0057	111
response to bacterium	4.18E-06	0.0057	18
single-multicellular organism process	5.01E-06	0.0064	109
positive regulation of response to external stimulus	6.18E-06	0.0068	13
regulation of inflammatory response	6.19E-06	0.0068	13
response to wounding	8.13E-06	0.0075	23
cell surface	8.02E-06	0.0075	25
regulation of response to wounding	8.20E-06	0.0075	16
receptor activity	1.01E-05	0.0088	26
regulation of developmental process	1.32E-05	0.0105	52
leukocyte migration	1.34E-05	0.0105	13
plasma lipoprotein particle	1.47E-05	0.011	5
high-density lipoprotein particle	1.86E-05	0.0133	4
extracellular region	2.12E-05	0.014	89
proteinaceous extracellular matrix	2.29E-05	0.014	15

regulation of response to external stimulus	2.16E-05	0.014	21
positive regulation of response to stimulus	2.30E-05	0.014	42
signal transducer activity	2.71E-05	0.0144	29
positive regulation of developmental process	2.56E-05	0.0144	30
molecular transducer activity	2.71E-05	0.0144	29
plasma lipoprotein particle organization	2.56E-05	0.0144	5
protein-lipid complex	3.45E-05	0.0178	5
regulation of multicellular organismal development	3.61E-05	0.018	41
regulation of immune system process	4.23E-05	0.0199	29
receptor binding	4.18E-05	0.0199	37
myeloid leukocyte migration	4.39E-05	0.0201	9
regulation of inflammatory response to antigenic stimulus	4.81E-05	0.0209	4
immune system process	5.20E-05	0.022	46
response to biotic stimulus	5.74E-05	0.0237	22
extracellular region part	5.91E-05	0.0237	83
rhythmic process	6.17E-05	0.0237	13
positive regulation of inflammatory response	6.16E-05	0.0237	7
complement activation	6.38E-05	0.0239	5
humoral immune response	6.57E-05	0.0241	7
scavenger receptor activity	7.37E-05	0.0247	5
cell communication	7.62E-05	0.0247	96
defense response to bacterium	7.00E-05	0.0247	9
response to external biotic stimulus	7.34E-05	0.0247	21
response to other organism	7.34E-05	0.0247	21
protein-lipid complex subunit organization	7.47E-05	0.0247	5
signaling	8.43E-05	0.0262	94
single organism signaling	8.43E-05	0.0262	94
hypersensitivity	9.56E-05	0.0272	3
acute inflammatory response	9.06E-05	0.0272	7
positive regulation of inflammatory response to antigenic stimulus	9.46E-05	0.0272	3
regulation of acute inflammatory response to antigenic stimulus	9.56E-05	0.0272	3
regulation of hypersensitivity	9.56E-05	0.0272	3
cell surface receptor signaling pathway	9.91E-05	0.0277	54
external side of plasma membrane	0.0001	0.0301	12
protein activation cascade	0.0001	0.0319	5
regulation of plasma lipoprotein particle levels	0.0001	0.032	6
positive regulation of biological process	0.0001	0.032	93
regulation of cell differentiation	0.0001	0.0355	38
cellular response to stimulus	0.0001	0.0357	106
leukocyte chemotaxis	0.0002	0.0415	9
lipid homeostasis	0.0002	0.0463	8
positive regulation of leukocyte chemotaxis	0.0002	0.0464	6
signal transduction	0.0002	0.0485	86
locomotion	0.0002	0.0497	35

DE transcripts were identified by RNA sequencing of left ventricular (LV) tissue from LUMKO and WT littermates (pools of n=3), using filters RPKM -fold difference <0.75 and >1.33 with p<0.001, yielding 714 DE transcripts (526 up- and 188 down-regulated). All (69) statistically significant categories with Benjamini-Hochberg (B-H) false discovery rate (FDR) <0.05 are shown with p-values and number (#) of molecules in the DE dataset overlapping with the total # of molecules in each GO category.

Table S6. KEGG pathway enrichment of differentially expressed transcripts in hearts in hearts of LUMKO 2 weeks post-AB

KEGG pathway	Overrepresented p-value	Multiple testing corrected p-value (FDR)	#DE transcripts in pathway
Staphylococcus aureus infection	*2.66E-10*	*5.95E-08*	*10*
Complement and coagulation cascades	*5.12E-08*	*5.74E-06*	*9*
Circadian rhythm - mammal	*2.37E-05*	*0.0018*	*6*
Systemic lupus erythematosus	*0.0005*	*0.0272*	*6*
Phagosome	0.0013	0.0566	10
Arachidonic acid metabolism	0.0063	0.2342	4
Cytokine-cytokine receptor interaction	0.0086	0.2767	7
Osteoclast differentiation	0.0107	0.3009	7
Drug metabolism - other enzymes	0.015	0.3458	3
ECM-receptor interaction	0.017	0.3458	5
Chagas disease (American trypanosomiasis)	0.0157	0.3458	6
Leukocyte transendothelial migration	0.0232	0.4328	6
Caffeine metabolism	0.0287	0.4696	1
Metabolism of xenobiotics by cytochrome P450	0.0293	0.4696	3
Prion diseases	0.0319	0.4762	3
Leishmaniasis	0.035	0.4898	4
Glutathione metabolism	0.041	0.5099	4
Drug metabolism - cytochrome P450	0.0405	0.5099	3

DE transcripts were identified by RNA sequencing of left ventricular (LV) tissue from LUMKO and WT controls (pools of n=3), using filters FPKM -fold difference <0.75 and >1.33 with p<0.001, yielding 714 DE transcripts (526 up- and 188 down-regulated). All (18) statistically significant categories (overrepresented p-value<0.05) are shown with p-values and number (#) of molecules in the DE dataset overlapping with the total # of molecules in each KEGG pathway. Benjamini-Hochberg (B-H) false discovery rate (FDR) multiple testing correction shows 4 KEGG pathways with p<0.05 (in *italic*).

Table S7. Assays for gene expression analysis

Assay	Mouse	Rat
SPON2	Mm00513596_m1	Rn01442950_m1
CILP2	Mm01349062_m1	Rn01420624_g1
PCNA	Mm00448100_g1	Rn01514538_g1
GDF-15	Mm00442228_m1	Rn00570083_m1
IL-6	Mm00446190_m1	Rn01410330_m1
IL-10	Mm00439614_m1	Rn01483988_g1
IL-1B	Mm00434228_m1	Rn00580432_m1
CSF2	Mm01290062_m1	Rn01456850_m1
LOX	Mm00495386_m1	Rn01491829_m1
COL1A2	Mm00483888_m1	Rn01526721_m1
COL3A1	Mm01254476_m1	Rn01437681_m1
FN1	Mm01256744_m1	Rn00569575_m1
ACTA2	Mm01546133_m1	Rn01759928_g1
POSTN	Mm01284919_m1	Rn01494627_m1
MMP2	Mm00439498_m1	Rn01538177_m1
TGFβ1	Mm01178820_m1	Rn00572010_m1
NPPA	Mm01255747_g1	Rn00664637_g1
NPPB	Mm01255770_g1	Rn00580641_m1
ACTA1	Mm00808218_g1	Rn01426628_g1
FMOD	Mm00491215_m1	Rn00589918_m1
LUM	Mm01248292_m1	Rn00579127_m1
TAGLN	Mm00441660_m1	Rn01642285_g1
RPL32	Mm02528467_g1	Rn00820748_g1
RPL4	Mm00834993_g1	Rn00821091_g1
HAS2	Mm00515089_m1	Rn00565774_m1
U6 snRNA	1973	1973
mmu-miR-21 / miR-21a-3p	2493	2493
hsa-mir-21 / miR-21a-5p	397	397

Pre-designed TaqMan assays used to determine gene expression by qPCR.

REFERENCES

1 Chakravarti, S. *et al.* Lumican regulates collagen fibril assembly: skin fragility and corneal opacity in the absence of lumican. *The Journal of cell biology* **141**, 1277-1286 (1998).

2 Bjørnstad, J. L. *et al.* A mouse model of reverse cardiac remodelling following banding-debanding of the ascending aorta. *Acta Physiologica* **205**, 92-102, doi:10.1111/j.1748-1716.2011.02369.x (2012).

3 Finsen, A. V. *et al.* Syndecan-4 is essential for development of concentric myocardial hypertrophy via stretch-induced activation of the calcineurin-NFAT pathway. *PloS one* **6**, e28302, doi:10.1371/journal.pone.0028302 (2011).

4 Melleby, A. O. *et al.* The Heparan Sulfate Proteoglycan Glypican-6 Is Upregulated in the Failing Heart, and Regulates Cardiomyocyte Growth through ERK1/2 Signaling. *PloS one* **11**, e0165079, doi:10.1371/journal.pone.0165079 (2016).

5 Strand, M. E. *et al.* Innate immune signaling induces expression and shedding of the heparan sulfate proteoglycan syndecan-4 in cardiac fibroblasts and myocytes, affecting inflammation in the pressure-overloaded heart. *The FEBS journal* **280**, 2228-2247, doi:10.1111/febs.12161 (2013).

6 Strand, M. E. *et al.* Shedding of syndecan-4 promotes immune cell recruitment and mitigates cardiac dysfunction after lipopolysaccharide challenge in mice. *Journal of molecular and cellular cardiology* **88**, 133-144, doi:10.1016/j.yjmcc.2015.10.003 (2015).

7 Christodoulou, D. C. *et al.* 5'RNA-Seq identifies Fhl1 as a genetic modifier in cardiomyopathy. *The Journal of clinical investigation* **124**, 1364-1370, doi:10.1172/jci70108 (2014).

8 Wang, Z., Gerstein, M. & Snyder, M. RNA-Seq: a revolutionary tool for transcriptomics. *Nature reviews. Genetics* **10**, 57-63, doi:10.1038/nrg2484 (2009).

9 Christodoulou, D. C., Gorham, J. M., Herman, D. S. & Seidman, J. G. Construction of normalized RNA-seq libraries for next-generation sequencing using the crab duplex-specific nuclease. *Current protocols in molecular biology* **Chapter 4**, Unit4.12, doi:10.1002/0471142727.mb0412s94 (2011).

10 Engebretsen, K. V. *et al.* Lumican is increased in experimental and clinical heart failure, and its production by cardiac fibroblasts is induced by mechanical and proinflammatory stimuli. *The FEBS journal* **280**, 2382-2398, doi:10.1111/febs.12235 (2013).
